# Sirt1 and Sirt3 Activation Improved Cardiac Function of Diabetic Rats via Modulation of Mitochondrial Function

**DOI:** 10.3390/antiox10030338

**Published:** 2021-02-24

**Authors:** Bugga Paramesha, Mohammed Soheb Anwar, Himanshu Meghwani, Subir Kumar Maulik, Sudheer Kumar Arava, Sanjay K Banerjee

**Affiliations:** 1 Non-Communicable Diseases (NCD), Translational Health Science and Technology (THSTI), Faridabad 121001, India; bparamesha@thsti.res.in (B.P.); soheb@thsti.res.in (M.S.A.); 2 APCER Life Sciences, US-PVG Department, New Delhi 110001, India; meghwani.himanshu@gmail.com; 3 Department of Pharmacology, All India Institute of Medical Sciences, New Delhi 110001, India; skmaulik@aiis.ac.in; 4 Department of Pathology, All India Institute of Medical Sciences, New Delhi 110001, India; aravaaiims@gmail.com; 5 Department of Biotechnology, National Institute of Pharmaceutical Research and Education, Guwahati 781001, India

**Keywords:** insulin resistance, oxidative stress, mitochondrial dysfunction, OXPHOS

## Abstract

In the present study, we aimed to evaluate the effect of Sirt1, Sirt3 and combined activation in high fructose diet-induced insulin resistance rat heart and assessed the cardiac function focusing on mitochondrial health and function. We administered the Sirt1 activator; SRT1720 (5 mg/kg, i.p.), Sirt3 activator; Oroxylin-A (10 mg/kg i.p.) and the combination; SRT1720 + Oroxylin-A (5 mg/kg and 10 mg/kg i.p.) daily from 12th week to 20th weeks of study. We observed significant perturbations of most of the cardiac structural and functional parameters in high fructose diet-fed animals. Administration of SRT1720 and Oroxylin-A improved perturbed cardiac structural and functional parameters by decreasing insulin resistance, oxidative stress, and improving mitochondrial function by enhancing mitochondrial biogenesis, OXPHOS expression and activity in high fructose diet-induced insulin-resistant rats. However, we could not observe the synergistic effect of SRT1720 and Oroxylin-A combination. Similar to in-vivo study, perturbed mitochondrial function and oxidative stress observed in insulin-resistant H9c2 cells were improved after activation of Sirt1 and Sirt3. We observed that Sirt1 activation enhances Sirt3 expression and mitochondrial biogenesis, and the opposite effects were observed after Sirt1 inhibition in cardiomyoblast cells. Taken together our results conclude that activation of Sirt1 alone could be a potential therapeutic target for diabetes-associated cardiovascular complications.

## 1. Introduction

Diabetes mellitus is a group of metabolic disorder characterized by hyperglycemia, caused by defects in insulin secretion, action, or both [1]. Worldwide the prevalence of diabetes is estimated to increase from 8.8% in 2017 to 9.9% by the year 2045 [2]. People suffering from diabetes mellitus are at increased risk for cardiovascular disease. Among them, 70% of the mortality is due to diabetes-associated cardiac complications. Hyperglycemia affects the structure and functions of the heart by several mechanisms like alteration of insulin signaling, lipid homeostasis, oxidative stress, mitochondrial function, formation of advanced glycogen end products (AGEs), Ca^++^ mishandling, increased inflammation, and increased utilization of fatty acids as an energy source [3,4]. As the heart is a continuous functioning organ, it needs a high amount of energy in the form of molecular ATP from mitochondria. So, the health of cardiac mitochondria plays an important role in the proper functioning of the heart. None of the current anti-diabetic drugs and adjuvant therapy such as ACE Inhibitors, SGLT2 inhibitors, DPP-IV inhibitors, and angiotensin receptor blockers are not effective to reduce cardiac complications in diabetic patients [5]. So, there is an urgent need to develop drugs to treat diabetes-associated cardiac complications.

As mitochondrial dysfunction and oxidative stress play a crucial role in diabetes-associated cardiac complications, by targeting the mitochondria, the diabetes-associated cardiac complications might be treated effectively. One of the mechanisms of perturbation of the physiological function of mitochondria is the post-translational modifications of mitochondrial proteins, mostly by acetylation [6]. Sirtuins are deacetylases proteins that moderate the acetylation status of proteins [1]. Among all seven sirtuins, nuclear sirtuin Sirt1 plays an important role in the regulation of cell metabolism and mitochondrial health by modulating the histones (H3A) and non-histone protein (NFκ-β) acetylation and thereby, regulating transcription of several crucial genes [7,8]. Sirt3 is a mitochondrial sirtuin and regulates fatty acid metabolism, TCA cycle, and oxidative phosphorylation and oxidative stress by deacetylating the mitochondrial proteins [9]. There are few reports which show the connection between nuclear and mitochondria communication, and its role in mitigating oxidative stress and improving mitochondrial function [10,11,12]. However, there is no report on Sirt1-Sirt3 axis that communicates between the nucleus and mitochondria and controls mitochondrial biogenesis and bio-energetic pathways in the diabetic heart. Therefore, the objective of the present study is to explore the role of Sirt1 and Sirt3 activation in mitochondrial metabolic pathways in the diabetic heart. We hypothesize that modulation of Sirt1 and Sirt3 protein activity independently or in a combination may improve mitochondrial function as well as diabetes-associated cardiac complications.

## 2. Material and Methods

### 2.1. Cell Culture

H9c2 cells (rat cardiomyoblast) were procured from ATCC (Manassas, VA, USA) and cultured under standard conditions that are 37 °C and 5% CO_2_ incubator (HERA CELL VIOS 160I, Thermo Scientific, Waltham, MA, USA). H9c2 cells were grown in DMEM media (Cat. No. SH30071.03, HyClone™, GE Healthcare Life Sciences, Hyclone laboratories, South Logan, UT, USA) having (4 mM L-glutamine and 4.5 gm/L L-glucose and sodium pyruvate) with 10% (*v*/*v*) Fetal bovine serum albumin (Cat. No. SH30071.03, HyClone™, GE Life Sciences, Hyclone laboratories, South Logan, UT, USA), penicillin and streptomycin mixture as antibiotic (Cat. No. SH30071.03, HyClone™, GE Life Sciences, Hyclone laboratories, South Logan, UT, USA).

#### 2.1.1. Treatment Conditions for Sirt1 Activation and Inhibition Study

H9c2 cells were treated with SRT1720 (Cat.no. A14367, Adooq bioscience, Barranca Parkway, Irvine, AB, Canada), a Sirt1 activator at a concentration of 0.25 µM, EX-527 (Cat. No. A10377, Adooq bioscience, Barranca Parkway, Irvine, AB, Canada), a Sirt1 inhibitor at a concentration of 50 µM; and DMSO as vehicle control. After 48 h of treatment, RNA and proteins were isolated for further analysis.

#### 2.1.2. Palmitate Induced Insulin Resistance in H9c2 Cells

10 mM palmitate stock solution was prepared by conjugating palmitate with fatty acid-free BSA (Cat No. A8806, Sigma Aldrich, St. Louis, MO, USA). Insulin resistance was induced in H9c2 cells with 200 µM of palmitate for about 24 h while control cells were treated with the same volume of BSA 20 µL/mL of media. The treatment groups were treated with (i) SRT1720, a Sirt1 activator (0.25 µM), (ii) Oroxylin-A, a Sirt3 activator (50 µM), (iii) SRT1720 + Oroxylin-A (0.25 µM and 50 µM) and (iv) Metformin, a standard antidiabetic drug (1 mM) along with 200 µM palmitate for about 24 h, DMSO was used as a vehicle. Throughout all the experiments, it was made sure that the final volume of the DMSO did not exceed 0.2% (*v*/*v*). Cells were subjected to starvation for about 2 h with low glucose media without serum for glucose uptake assay. H9c2 cells were incubated with a fluorescent compound 2-NBDG (Cat. No. N13195, Waltham, MA, USA), a glucose analog, at a concentration of 100 µM and insulin 100 nM (Cat. No. I0516, Sigma Aldrich, St. Louis, MO, USA). After 30 min of incubation cells were lysed with 0.5% (*v*/*v*) Triton-X100 (Cat. No. TB0198, Bio Basic 20 Konard crescent, Markham, ON, Canada) and florescence was measured at 464/540 nm by using a multi-plate reader (Molecular Devices, Spectra max M2^e^, San Jose, CA, USA).

#### 2.1.3. Cellular-ROS Measurement

Cellular reactive oxygen species were measured by BD FACS Canto^TM^ II (BD Biosciences, Piscataway, NJ, USA). Briefly, following 24 h of previously mentioned treatments, 10 µM of 2′, 7′-dichlorodihydrofluorescein diacetate (Cat. No. D399, Invitrogen, San Diego, CA, USA) is incubated for 30 min at 37 °C. Cells were trypsinized with Trypsin-EDTA (Cat. No. CC5027.010L, Cell Clone^TM^) and centrifuged at 1100 rpm for 3 min followed by three times PBS washing. Cells were suspended in filtered 1× PBS and samples were acquired under the Alexa-488-channel. The data was analyzed by FlowJo^TM^ software for Windows Version V10, Ashland.

#### 2.1.4. Crude Mitochondrial-ROS Measurement

Crude mitochondrial reactive oxygen species were measured with Mito-Sox red (Cat. No. M36008, Thermo Fisher Scientific, Waltham, MA, USA). Briefly, after completion of the above mentioned treatment period, cells were stained with Mitosox-red dye at a concentration of 5 µM for about 30 min at 37 °C. Cells were trypsinized and washed three times with 1× PBS. Cells were suspended in filtered 1× PBS solution and acquired by BD FACS Canto-II under PE-channel and data was analyzed by FlowJo^TM^ software.

#### 2.1.5. Mitochondrial Content

Mitochondrial content was measured with Mit-tracker green-FM (Cat. No. M7514, Thermo Fisher Scientific, Waltham, MA, USA). Briefly, after completion of the above-stated treatment period, cells were stained with mito-tracker green-FM dye at a concentration of 200 nM for about 20 min at 37 °C. Cells were trypsinized and washed three times with 1× PBS. Cells were suspended in filtered 1× PBS solution and acquired by BD FACS Canto-II under FITC-channel and data was analyzed by FlowJo^TM^ software.

#### 2.1.6. Mitochondrial Membrane Potential

The mitochondrial intermembrane potential was measured with tetramethylrhodamine methyl ester (TMRM Cat. No. T668, Invitrogen, San Diego, CA, USA). Briefly, after completion of the earlier mentioned treatment period, cells were incubated with TMRM dye at a concentration of 100 nM for about 10 min at 37 °C. Cells were trypsinized and washed with 1× PBS three times. Cells were suspended in filtered 1× PBS solution, and acquired by BD FACS Canto-II under PE-channel and data was analyzed by FlowJo^TM^ software.

#### 2.1.7. Evaluation of Mitochondrial Oxygen Consumption Rate

An extracellular flux analyzer (XFe24, Seahorse Biosciences, Bellarica, Massachusetts, MA, USA) was used to assess mitochondrial oxygen consumption rate as described previously [13,14]. H9c2 cells were seeded at a density of 3 × 10^4^ cells/well. In brief, after 24 h treatment, the media was replaced by basal media (Seahorse XFe flux analyzer compatible) deprived of serum and glucose. Measurements of oxygen concentration were periodically made every 4 min over 150 min, and the rate of oxygen consumption was obtained from the slopes of concentration change versus time. Oligomycin (1 μM), carbonyl cyanide-4-(Trifluoro methoxy) phenyl hydrazone (FCCP; 2 μM), rotenone, and antimycin-A (1 μM each) were preloaded into the cartridge. Baseline rates were measured four times. Oligomycin, FCCP, rotenone, and antimycin-A were injected into the wells as per the protocol. Post-exposure of the OXPHOS uncoupler and the inhibitors, oxygen consumption rate measurements were made four times. Maximal respiration was defined as the FCCP-induced oxygen consumption rate minus the rotenone and antimycin-A inhibited oxygen consumption rate. Spare respiratory capacity was defined as the FCCP-induced oxygen consumption rate minus the oxygen consumption rate at baseline. ATP-linked oxygen consumption rate was calculated as the baseline oxygen consumption rate minus the oligomycin-inhibited oxygen consumption rate.

#### 2.1.8. mRNA Expression Studies by RTPCR

Total RNA was isolated from cardiomyoblast using TRI reagent (Sigma Aldrich, St. Louis, MO, USA) as per the manufacturer’s protocol and the RNA quantification was done by using nanodrop (Thermo Fisher Scientific, Waltham, MA, USA). DNase treatment was done for the RNA sample by using DNase enzyme (Epicentre, Lane lindenhurts, IL, USA). 1 μg total RNA has been used for cDNA preparation. RT-PCR was performed by using the Emerald Amp GT PCR master mix (RR310A, Takara, Kusatsu, Shiga, Japan). The list of primers used for PCR was mentioned in Table S1.

#### 2.1.9. Protein Expression

After completion of treatment, cells were washed with 1× PBS, cells were lysed with RIPA buffer having protease and phosphatase inhibitor. Cell lysates were subjected to centrifugation at 4 °C about 25 min followed by supernatant was collected and protein quantification was done by using the BCA kit (Cat No. 2327, Thermo Scientific, Waltham, MA, USA). Western blotting was done by loading 40 μg of protein samples into 10% (*v*/*v*) SDS-PAGE and protein bands were transferred onto the PVDF membrane. After transferring the proteins on to the PVDF membrane were washed with TBST buffer three times followed by membranes were incubated overnight at 4 °C with primary antibodies. After completion of incubation with a primary antibody, blots were washed with TBST buffer for three times, and membranes were incubated with secondary antibody for 1 h at room temperature. The protein band signal was acquired by Biorad-Gell Doc using Luminata Forte Western HRP Substrate (Merck Millipore; Cat No. WBLUF0100, Billerica, MA, USA). Primary and secondary antibodies were used from Abcam and cell signaling for western blotting. The list of primary and secondary antibodies used for the study was mentioned in Table S2.

### 2.2. In-Vivo Study

Animal studies were performed as per the standard operating procedures (SOP) of the Institutional Animal Ethical Committee (IAEC/THSTI/2017-14) of the Translational Health Science and Technology Institute (THSTI), Faridabad. Male SD rats weighing about 150 to 200 g and 6–8 weeks old were purchased from the National Institute of Pharmaceutical Education and Research (NIPER), SAS Nagar, Mohali, Punjab, India. Insulin resistance (pre-diabetes) was induced in SD rats by feeding with high fructose diet 65% Kcal from fructose (Cat. No. D11707R, Research diets, New Brunswick, NJ, USA). Control animals were fed with corn starch diet (Cat. No. D11708B, Research diets, New Brunswick, NJ, USA) from the research diet composition of the used diets were shown in Table S3. The induction of insulin resistance was confirmed by monitoring fasting blood glucose and intraperitoneal glucose tolerance test (IPGTT). After confirming the insulin resistance, control and high fructose diet fed animals were treated with (i) vehicle (PEG400 and Tween-80 at a ratio of 90:10), (ii) SRT1720 (a Sirt1 activator) 5 mg/kg/day, i.p., (iii) Oroxyin-A (a Sirt3 activator) 10 mg/kg/day, i.p., (iv) SRT1720 5 mg/kg/day + Oroxylin-A 10 mg/kg/day, i.p. and (v) metformin (a standard antidiabetic drug) 300 mg/kg/day, p.o. for 8 weeks (i.e., 12 to 20 weeks). After eight weeks of treatment intraperitoneal glucose tolerance test, blood pressure, ECG and echocardiography have performed and blood samples were collected from all experimental animals for the evaluation of serum parameters. Then, the animals were sacrificed under anesthesia (isoflurane) followed by cervical dislocation and heart tissues were collected and stored at −80 °C for downstream analysis.

#### 2.2.1. Intraperitoneal Glucose Tolerance Test

The Intraperitoneal glucose tolerance test was performed on overnight (~12 h) fasted animals by injecting 2 gm/kg (*w*/*w*) of D-glucose solution through the intraperitoneal route. Blood glucose levels were measured at different time points 0, 15, 30, 60, and 120 min and the area under the curve was calculated. Glucose tolerance of the corn-starch and high fructose diet-fed animals were performed at 12th week of the study. Similarly, IPGTT was performed in control, high fructose diet-fed animals and treatment groups at 20th week, before sacrificing the animals. The area under the curve of blood glucose was calculated and plotted.

#### 2.2.2. Electrocardiography

Electrocardiography of the rats was performed under anesthesia with ketamine (50 mg/kg) and Xylazine (10 mg/kg) at two-time points i.e., 12th week and 20th week. ECG was recorded for about 10 min with power lab system from AD instruments, (Colorado springs, CO, USA) and data was analyzed.

#### 2.2.3. Blood Pressure

The blood pressure of the rats was monitored at two different time points i.e., 12th and 20th week of the study. Before recording the blood pressure of the experimental animals, we habituated them for restrainers for about ten days and then blood pressure was recorded by using a Non-Invasive Blood Pressure System for Rodents (LE5002; Harvard instruments, Liobregat, Barcelona, Spain).

#### 2.2.4. Echocardiography

Rats were subjected to echocardiography at two different time points i.e., 12 (before starting the treatment) and 20 weeks before sacrificing the animals. Following anesthesia with ketamine (50 mg/kg BW, i.p.) and Xylazine (10 mg/kg BW, i.p.), chest hair was removed and rats were examined in the supine position with the transducer probe placed gently in the left parasternal position. Hemodynamic parameters of the experimental animals were evaluated by two-dimensional M-mode echocardiography with a 10–11.5 MHz neonatal cardiac probe transducer of high frame rate and shallow focus (10–25 mm) from a short-axis view at the level of the papillary muscles of left ventricle (LV) using a Philips 2D echocardiography machine. Posterior wall (PW) and intraventricular septum (IVS) thicknesses were recorded in diastole and systole. Left ventricular end-diastolic dimensions (LVD_d_) and left ventricular dimension in systole (LVD_s_) were recorded from the short-axis view at the level of papillary muscle from the trailing edge of septum to the leading edge of posterior wall muscles. The images, each composed of five to nine consecutive heart cycles, were digitally transferred online to a computer, and subsequently analyzed by an analyst blinded to the treatment groups. Three representative cycles were analyzed and averaged. Left ventricular diameter during systole and diastole, posterior wall thickness of left ventricle during systole and diastole was measured by using PmsDview _2.1 software, and ejection fraction (EF), and fractional shortening (FS) was calculated as per standard methods.

#### 2.2.5. Serum Lipid Profile

Rats were anesthetized by using Isoflurane and blood was collected through retro-orbital puncture method. Lipid profile i.e., triglycerides, LDL, HDL, total cholesterol were measured in serum with Auto-analyzer (EXL-200 from seimense) by using commercially available kits from Siemens, Newark, NJ, USA.

#### 2.2.6. Serum Free Fatty Acids

Serum-free fatty acid levels were measured using a commercially available kit (Randox: cat No. FA115, Banglore, Karnataka, India). Fasting serum free fatty acid levels were measured according to the manufacturer’s protocol.

#### 2.2.7. Serum Insulin

Serum insulin levels were measured by using a commercially available kit from Crystal Chem (Cat. No. 90010, Elk Grove Village, IL, USA). Measurement of fasting serum insulin was performed according to the manufacturer’s protocol. HOMA-IR was calculated to assess insulin resistance using the following formula HOMA-IR = Fasting serum insulin (IU)*Fasting blood glucose levels(mg/dL)/405 [15].

#### 2.2.8. mRNA Expression Studies by Real-Time PCR

Frozen heart tissues were washed with PBS, cut into small pieces and RNA was isolated by using Trizol method. RNA quality and quantities were measured by the nanodrop (Thermo scientific, Waltham, MA, USA). Then the RNA was subjected to DNase treatment. cDNA was synthesized by using DNase treated RNA samples and mRNA levels were measured by real-time PCR using FastStart Essential DNA green master (SYBR, Roche) using light cycler 96-well (Roche, Mannheim, Germany). mRNA levels were normalized using RPL32 mRNA as a reference. Gene-specific primers and PCR cycling conditions were as follows: Denaturation for 10 min at 95 °C; followed by 45 cycles of 3 step amplification method (10 s at 95 °C denaturation, 10 s Annealing at 60 °C, 10 s extension at 72 °C) and the data was analyzed by 2(-Delta Delta C(T)) method. The list of primers used for PCR was mentioned in Table S1.

#### 2.2.9. Protein Expression

Heart tissues were minced into small pieces and homogenized with RIPA buffer added protease and phosphatase inhibitor). Tissue homogenates were subjected to centrifugation at 12,000 rpm for 25 min at 4 °C and the supernatant was collected and Protein quantification was done by using the BCA kit (Cat No. Cat.No.2327, Waltham, MA, USA). Western blotting was done by loading 30 μg of protein samples into 10% (*v*/*v*) SDS-PAGE and protein bands were transferred onto the PVDF membrane. Then PVDF membranes were incubated with primary antibodies (Abcam and cell signaling) followed by HRP-conjugated secondary antibodies then blots were developed. The list of primary and secondary antibodies used for the study was mentioned in Table S2.

#### 2.2.10. Mitochondrial Protein Enrichment

Mitochondrial protein enrichment was done from the heart tissue samples by using a mitochondrial isolation kit from Thermo fisher scientific (Cat. No. 89801, Waltham, MA, USA). Samples were used for the evaluation of SIRT3 activity [16,17].

#### 2.2.11. Mitochondrial DNA Isolation

50 mg of frozen heart tissue samples were homogenized in 1 mL of freshly prepared buffer containing 100 mM Tris-HCl, 10 mM EDTA and 250 mM sucrose. Nuclei and cellular debris were separated by centrifugation at 1500× *g* for 15 min at 4 °C. The supernatant was collected and centrifuged at 10,000 rpm for 10 min at 4 °C to pellet down the mitochondria. Further, denaturation and complete solubilization of proteins of mitochondrial pellets was done by treating the above-obtained pellet with 500 μL of buffer containing 10 mM Tris HCl (pH 7.6), 10 mM KCl, 10 mM MgCl_2_, 0.4 M NaCl and 2 mM EDTA, and 80 μL of 10% (*w*/*v*) SDS. This mixture was incubated overnight at 55 °C. From the solubilized protein mixture, desalting was done by adding 200 μL of 6 M NaCl and centrifuged for 30 min at 11,300× *g*. Mitochondrial DNA is precipitated by adding twice the volume of earlier obtained supernatant with absolute ethanol. Finally, the mitochondrial DNA pellet is washed twice with 70% ethanol and air-dried. Finally, the dried mitochondrial DNA pellet dissolved in 100 μL of molecular biology grade water. DNA quantity and purity were measured by NanoDrop spectrophotometer. To quantify mitochondrial DNA copy number, q-PCR was done with mitochondrial encoded genes, Cytochrome-C oxidase-II (CO-II) and the data were normalized by nuclear-encoded gene β2-microglobulin (β2M) whose expression remains constant [18].

#### 2.2.12. Nuclear Isolation

Nuclear isolation was done from the heart tissue samples by using a nuclear extraction kit from Signosis (Cat.no.SK-0001, Santa clara, CA, USA). Samples were used for the evaluation of SIRT1 activity.

#### 2.2.13. Histopathology Studies

Heart tissues fixed in neutral buffer formalin (10%) (*v*/*v*) were paraffin-embedded for histopathological analysis. Sections (5-µm) were stained with hematoxylin and eosin and masons trichrome for further analysis.

### 2.3. Biochemical Assays

For biochemical analysis of heart tissue samples were prepared by using 1× PBS buffer. Protein quantification was done using a BCA kit.

#### 2.3.1. Evaluation of Sirt1 Activity

Evaluation of Sirt1 activity was done by a commercially available kit from Cyclex (Cat. No. CY-1151 V2). The assay was performed according to the manufacturer’s protocol. Sirt1 activity was measured by incubating nuclear extract with a fluorescent-labeled protein and NAD+ (co-substrate of SIRTs) at 37 °C for about 30 min then fluorescence of the sample was measured at 360/460 nm (Excitation/emission wavelength) after adding developing solution.

#### 2.3.2. Evaluation of Sirt3 Activity

Determination of Sirt3 activity was performed by a commercially available kit from Cyclex (Cat. No. CY-1153 V2). Sirt3 activity was determined by incubating mitochondrial extract along with a fluorescent-labeled protein and NAD+ (co-substrate of SIRTs) at 37 °C for about 30 min. The fluorescence of the sample was measured at 360/460 nm (Excitation/emission wavelength) after the addition of the developing solution.

#### 2.3.3. Antioxidant Parameters

##### SOD Activity

SOD activity was evaluated by a commercially available kit from Sigma-Aldrich (Cat. No. 19160, St. Louis, MO, USA).

##### Catalase

Myocardial catalase activity was measured using the method described by Beers and Sizer. The decomposition of H_2_O_2_ was measured for about 3 min with 30-sec interval using spectrophotometer at 240 nm in presence of catalase [19].

##### Estimation of GSH

Reduced glutathione is estimated by Ellman’s method. Ellman’s reagent DTNB (5-5′-dithiobis-(2-nitrobenzoic acid)) reacts with sample containing thiol groups and gives absorbance at 412 nm by using spectrophotometer [20].

##### TBARS

The extent of lipid peroxidation in the heart was evaluated by measuring malondialdehyde (MDA) content in the tissue lysate according to the modified method [21] depends on the reaction with thio-barbituric acid. Data were represented as nanomoles (nM) per gm heart weight using an excitation co-efficient of 1.5 × 10^5^ M^−1^ cm^−1^.

### 2.4. Mitochondrial Enzymatic Assays

#### 2.4.1. Citrate Synthase

Citrate synthase activity was evaluated according to Anthony et al. [22]. Briefly, 0.6 mm oxaloacetate was added to the assay containing 5,5-dithio-bis 2-nitrobenzoic acid (DTNB). The reaction was initiated after adding acetyl-CoA. The change in absorbance was measured at 412 nm for about 10 min with a 10-sec interval [22].

#### 2.4.2. β-Hydroxy acyl CoA Dehydrogenase

Beta hydroxy acyl CoA dehydrogenase is a mitochondrial enzyme, which is involved in fatty acid metabolism. Beta hydroxy acyl CoA dehydrogenase activity was measured as per the protocol described by Civitarese [22]. Briefly, after an initial normalization of the buffer containing triethanolamine, EDTA, and NADH at 340 nm, acetyl CoA was added, and absorbance was measured for 5 min with 10 sec interval.

#### 2.4.3. NADH Dehydrogenase

The activity of NADH dehydrogenase was determined as described by King and Howard. Mitochondrial preparation was added to the reaction mixture containing 6 mM NADH, 0.2 M glycyl-glycine (pH 8.5), 0.02 M NaHCO_3_, and 1 mM oxidized Cytochrome-C. NADH dehydrogenase catalyzed reduction of cytochrome-c, and the change in absorbance was followed by spectrophotometrically at 550 nm for 5 min with 10 sec interval [23].

#### 2.4.4. Succinate Dehydrogenase

Succinate dehydrogenase, a mitochondrial enzyme of ETC complex-II, converts succinate to fumarate. The assay is based on the change in the absorbance of Cytochrome-C by the spectrophotometric method at 550 nm for 5 min with 10 sec interval.

#### 2.4.5. Cytochrome-C Oxidase

Cytochrome-C oxidase enzyme, a part of the electron transport chain complex, present in the mitochondrial intermembrane was measured using reduced Cytochrome-C and sodium hydrosulfite as described previously [22]. The reaction was started by adding sample to the reaction mixture and the change in sample absorbance was measured at 550 nm for 5 min at 10-s interval [22].

##### Statistical Analysis of Data

All the data was analyzed by one-way ANOVA (analysis of variance) followed by Bonferroni’s post hoc comparisons tests were performed for the experiments which were having more than two groups and experiments which were having two groups were analyzed by two-tailed *t*-test. Results were represented as Mean ± SEM.

## 3. Results

### 3.1. Sirtuin Activation Reduces Insulin Resistance in High Fructose Diet-Induced Pre-Diabetic Rats and Palmitate-Treated Cardiomyoblast Cells

**Figure 1:**
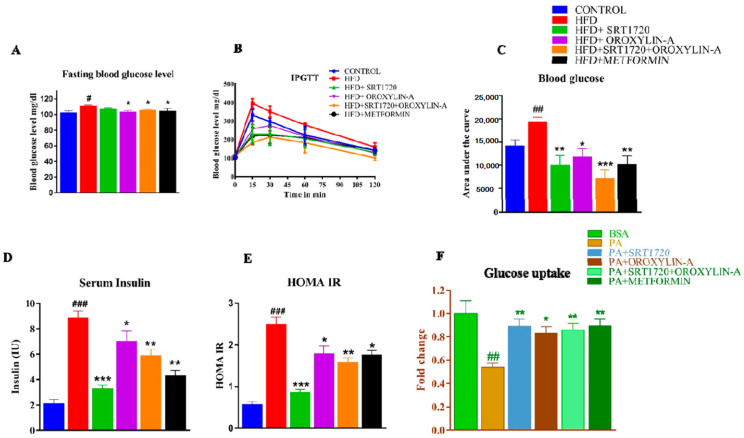
Sirtuin activation reduces insulin resistance in rats fed with high fructose diet and palmitate treated cardiomyoblast (H9c2) cells. (**A**) Fasting blood glucose levels. (**B**) Intraperitoneal glucose tolerance test. (**C**) Area under the curve of blood glucose levels. (**D**) Fasting serum insulin levels. (**E**) HOMA-IR. (**F**) Glucose uptake in palmitate treated cardiomyoblast (H9c2) cells. Data was represented as Mean ± SEM, ^#^ *p* < 0.05 vs. Control, ^##^ *p* < 0.01 vs. Control, ^###^ *p* < 0.001 vs. Control, * *p* < 0.05 vs. HFD, ** *p* < 0.01 vs. HFD, *** *p* < 0.001 vs. HFD, (*n* = 5). Glucose uptake assay data was represented as Mean ± SEM, ^##^ *p* < 0.01 vs. BSA, * *p* < 0.05 vs. PA, ** *p* < 0.01 vs. PA, (*n* = 3).

High fructose diet (HFD) significantly (*p* < 0.05) increased fasting blood glucose, insulin-resistance, serum insulin levels, and HOMA-IR in high fructose diet-fed (HFD) rats when compared with control diet-fed rats. Sirtuin activation (SRT1720, Oroxylin-A, and combination of SRT1720 and Oroxylin-A) in HFD fed rats significantly (*p* < 0.05) reduced the fasting blood glucose, insulin resistance, serum insulin, and HOMA-IR levels. The change observed after sirtuin activation was compared with highfructose diet fed rats (Figure 1A–E). The effect of sirtuin activation on insulin resistance was further validated by performing an in-vitro 2-NBDG uptake assay on palmitate-induced insulin-resistant cardiomyoblast (H9c2) cells. We found improvement in glucose uptake after sirtuin activation in palmitate-induced insulin-resistant cardiomyoblast (H9c2) cells (Figure 1F).

### 3.2. Sirtuin Activation Reduces Serum Lipid Profile in High Fructose Diet-Fed Rats

Most of the serum lipid parameters (triglycerides, LDL, total cholesterol and free fatty acids) were significantly (*p* < 0.05) increased in high fructose diet-fed (HFD) rats when compared to the corn-starch diet-fed (Control) rats. Sirtuin activation with SRT1720, Oroxylin-A, and combination of SRT1720 and Oroxylin-A in HFD rats significantly (*p* < 0.05) reduced elevated serum triglyceride levels. While Sirt1 activation and combination showed significant (*p* < 0.05) reduction of elevated serum cholesterol, triglycerides, free fatty acids and LDL levels, Sirt3 activation and metformin improved serum triglyceride, free fatty acids and HDL levels in HFD rats (Table 1).

### 3.3. Sirtuin Activation Improves Structural and Functional Characteristics of Heart in High Fructose Diet-Fed Rats

**Figure 2:**
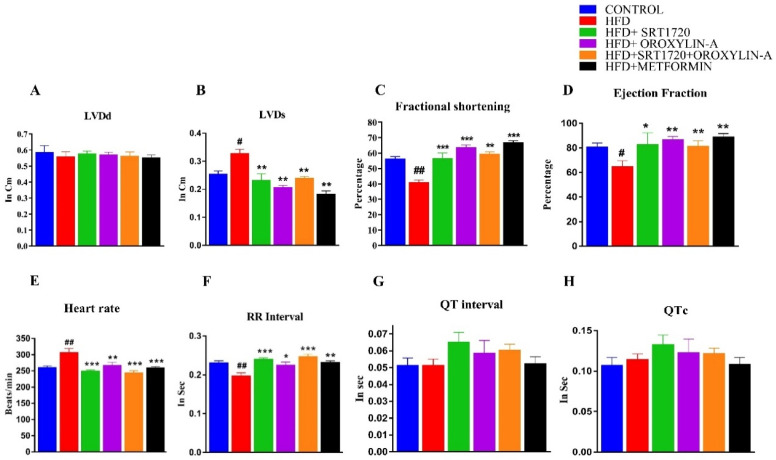
Sirtuin activation normalizes Echocardiography (**A**–**D**) and ECG (**E**–**H**) abnormalities in rats fed with high fructose diet. (**A**) Left ventricular internal diameter during diastole (LVD_d_). (**B**) Left ventricular internal diameter during systole (LVD_s_). (**C**) Fractional shortening. (**D**) Ejection fraction. (**E**) Heart rate. (**F**) RR-interval. (**G**) QT-interval. (**H**) QTc-interval. Data was represented as Mean ± SEM, ^#^ *p* < 0.05 vs. Control, ^##^ *p* < 0.01 vs. Control, * *p* < 0.05 vs. HFD, ** *p* < 0.01 vs. HFD, *** *p* < 0.001 vs. HFD, (*n* = 5).

High fructose diet feeding about 12 weeks significantly (*p* < 0.05) increases posterior wall thickness and heart weight to body weight ratio when compared to control diet-fed rats. Feeding of high fructose diet for 12-weeks did not alter the rest of the structural and functional parameters of the heart (Figure S1A–H). 20 weeks high fructose diet feeding to rats (HFD) significantly (*p* < 0.05) perturbs the structural (left ventricular lumen diameter during systole (LVDs)) and functional (ejection fraction (EF) and fractional shortening (FS)) characteristics of diabetic rat heart. High fructose diet did not affect the left ventricular lumen diameter during diastole (LVDd) when compared to control diet-fed rats. Administration of SRT1720, Oroxylin-A and combination of SRT1720 and Oroxylin-A for about 8 weeks (from 12 to 20 weeks) significantly (*p* < 0.01) improved structural (left ventricular lumen diameter during systole (LVDs)) and functional parameters (ejection fraction and fractional shortening) of pre-diabetic rat heart induced by high fructose diet. The standard drug, metformin treatment also showed improvement of all of the above structural and functional parameters (*p* < 0.01) (Figure 2A–D).

### 3.4. Sirtuin Activation Corrected Perturbed ECG Parameters in with High Fructose Diet-Fed Rats

High fructose diet feeding significantly (*p* < 0.05) increased ECG parameters i.e., heart rate and RR-interval of rats. Sirtuin activation with SRT1720, Oroxylin-A, and combination of SRT1720 and Oroxylin-A from 12 to 20-weeks significantly (*p* < 0.05) decreased the high fructose diet-induced increase in the heart rate and RR-intervals. Administration of metformin in rats fed with high fructose diet (HFD) also significantly (*p* < 0.05) decreased heart rate and increased RR-interval. We have not observed any significant change of QT and QTc-interval parameters in any of the groups (Figure 2E–H).

### 3.5. Sirtuin Activation Reduces Elevated Blood Pressure in High Fructose Diet-Fed Rats

High fructose diet significantly (*p* < 0.05) increased systolic blood pressure at 12 week time but did not show any significant changes in other parameters like diastolic and mean arterial blood pressure (Figure S2A). 20 weeks high fructose diet (HFD) feeding significantly (*p* < 0.01) increased systolic blood pressure when compared to control diet-fed rats. Although we observed increased diastolic and mean arterial blood pressure after 20-weeks of high fructose diet feeding (HFD), those changes were not significant. However, chronic sirtuin activation treatment (from 12 to 20 weeks) able to reduce increased systolic and mean arterial blood pressure significantly (*p* < 0.05) when compared to HFD fed rats (Figure S2B). Administration of standard drug metformin also reduced systolic and mean arterial blood pressure significantly (*p* < 0.05) when compared to HFD fed rats.

### 3.6. Sirtuin Activation Reduces Cardiac Fibrosis in High Fructose Diet-Fed Rats

**Figure 3:**
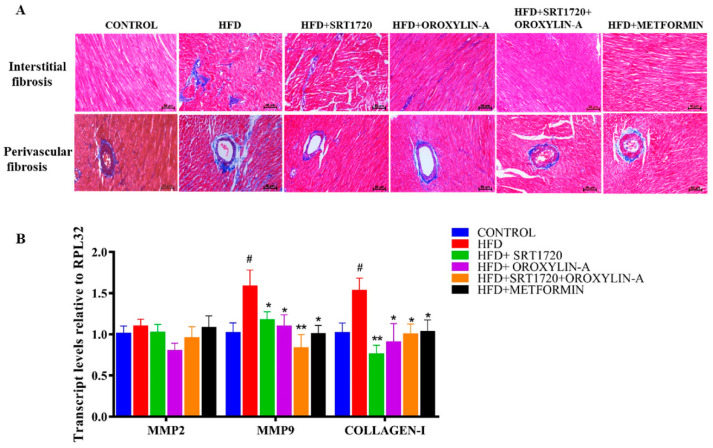
Sirtuin activation attenuates high fructose diet-induced cardiac fibrosis in rats. (**A**). Histopathology images after Masson trichrome staining (upper panel represents interstitial fibrosis and lower panel represents perivascular fibrosis). (**B**) mRNA expression levels of fibrotic Genes. Data was represented as Mean ± SEM, ^#^ *p* < 0.05 vs. Control, * *p* < 0.05 vs. HFD, ** *p* < 0.01 vs. HFD, (*n* = 5 for mRNA expression study and *n* = 3 for histological studies).

High fructose diet-feeding increases interstitial and perivascular cardiac fibrosis in rats. The same was confirmed by histopathological examination by Masson trichome staining and measuring pro-fibrotic gene (mRNA) expression levels. High fructose diet increased interstitial and perivascular fibrosis of cardiac tissue and significant (*p* < 0.05) increase in the pro-fibrotic gene (MMP9 and collagen-1) mRNA expression levels in HFD fed rats when compared to control rats. The cardiac tissue fibrosis and pro-fibrotic gene expression level were significantly (*p* < 0.05) reduced with sirtuin activators SRT1720, Oroxylin-A, a combination of SRT1720 and Oroxylin-A, and standard drug metformin treatment (Figure 3A,B).

### 3.7. Sirtuin Activation Reduces Fetal Cardiac Hypertrophic Gene Expression in High Fructose Diet-Fed Rats

We assessed the cardiac hypertrophy in HFD fed rats by morphometric parameters like heart weight to body weight and heart weight to tail length ratio. Although we observed a significantly increased heart weight to body weight ratio after,12 weeks of high fructose diet feeding (Figure S1A), we did not observe any significant changes at the end of the study (Figure S3A,B). However, we found a significant increase in fetal hypertrophic gene β-MHC mRNA expression in HFD fed rats at 20th week. High fructose diet did not affect the expression of other fetal hypertrophic genes (ANP and BNP) mRNA expression when compared to control rats. The cardiac hypertrophic gene β-MHC (mRNA) expression level was significantly (* *p* < 0.05) reduced by sirtuin activation in HFD fed rat hearts (Figure S3C). We also evaluated the body weight change at 12th and 20th week of the study. We observed a decrease in the bodyweight of the high fructose diet fed animal at 12th and 20th week time point but, the changes were not significant when compared to control diet fed animals. We observed a significant decrease in body weight of SRT1720 and Srt1720 + oroxylin-A treated groups when compared to control diet-fed animals (Figures S1B and S3D).

### 3.8. Sirtuin Activation Increases the Cardiac Expression and Activity of Sirt1 and Sirt3 in High Fructose Diet-Fed Rats

**Figure 4:**
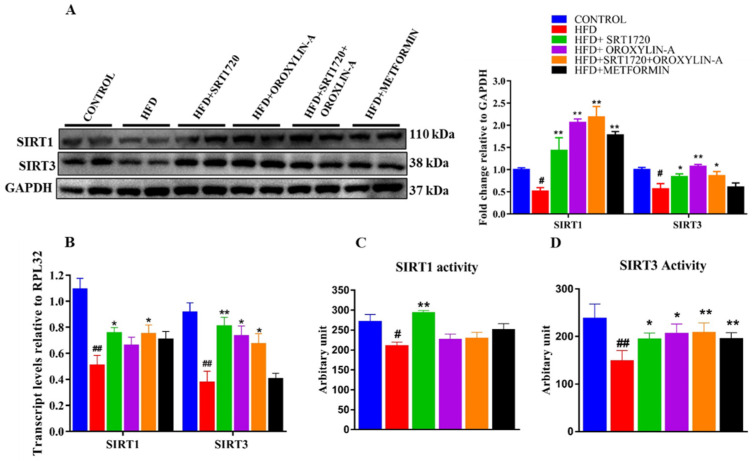
Sirtuin activation enhances the cardiac expression and activity of Sirt1 and Sirt3 in the pre-diabetic rats fed with high fructose diet. (**A**) Sirt1 and Sirt3 protein expression. (**B**) Sirt1 and Sirt3 mRNA expression. (**C**) Sirt1 enzymatic activity. (**D**) Sirt3 enzymatic activity. Data was represented as Mean ± SEM, ^#^ *p* < 0.05 vs. Control, ^##^ *p* < 0.01 vs. Control, * *p* < 0.05 vs. HFD, ** *p* < 0.01 vs. HFD, (*n* = 4 for western blotting, *n* = 5 for mRNA expression and enzymatic activity) (Blotts were developed different gels due to close molecular weight of the protein of interest, an equal amount of the proteins were loaded into the gels).

We measured the cardiac expression and activity of Sirt1 and Sirt3 in HFD fed rats, and we found a significant (*p* < 0.05) decreasedexpression of Sirt1 and Sirt3 in HFD fed rats when compared to control rats. However, activation of sirtuins with SRT1720, Oroxylin-A, and combination of SRT1720 and Oroxylin-A in high fructose diet-fed rats significantly (*p* < 0.05) enhanced the cardiac expression and activity of Sirt3 when compared to HFD fed rats but Sirt1 activity was improved only in SRT1720 treated animals when compared to high fructose diet-fed animals. However, metformin administration to high fructose diet-fed rats did not enhance the cardiac mRNA expression of both Sirt1 and Sirt3 (Figure 4B), and activity of Sirt1 (Figure 4C) when compared to HFD fed rats.

### 3.9. Sirtuin Activation Improves Cardiac Mitochondrial Biogenesis in High Fructose Diet-Fed Rats and Palmitic Acid (PA) Induced Insulin Resistant Cardiomyoblast Cells

**Figure 5:**
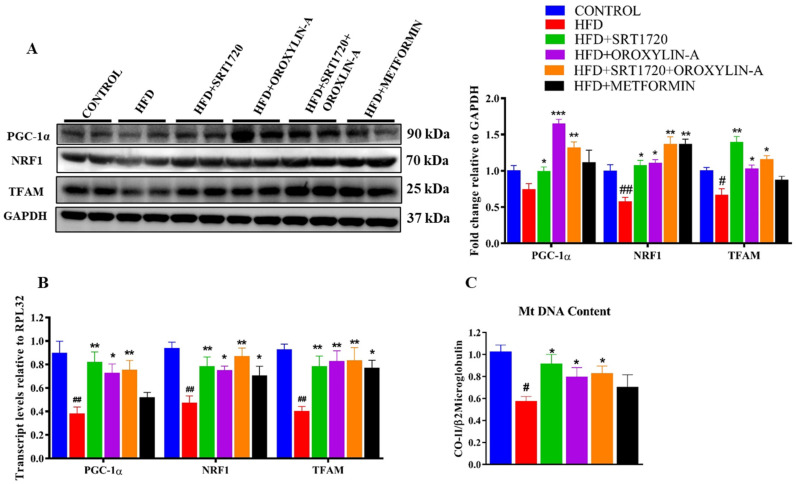
Sirtuin activation enhances mitochondrial biogenesis in the heart of high fructose diet-induced pre-diabetic rats. (**A**) Mitochondrial biogenesis related transcription factors protein expression. (**B**) Mitochondrial biogenesis-related transcription factors mRNA expression levels. (**C**) Mitochondrial DNA content. Data was represented as Mean ± SEM, ^#^ *p* < 0.05 vs. Control, ^##^ *p* < 0.01 vs. Control, * *p* < 0.05 vs. HFD, ** *p* < 0.01 vs. HFD, *** *p* < 0.001 vs. HFD, (*n* = 4) for western blotting, (*n* = 5) for mRNA expression, mitochondrial-DNA content.

Cardiac mitochondrial biogenesis was evaluated in rats from all groups. High fructose diet significantly (*p* < 0.05) decreased the mitochondrial biogenesis related gene and protein (PGC-1α, NRF1 and TFAM) expression when compared to control diet-fed rats. However, SRT1720, Oroxylin-A, and combination of SRT1720 and Oroxylin-A significantly (*p* < 0.05) improved NRF1 and TFAM mRNA and protein expression levels. PGC-1α expression was significantly increased in Oroxylin-A and SRT1720 + Oroxylin-A treated animals only when compared to high fructose diet fed animals. Metformin administration significantly (*p* < 0.05) improved NRF1 (both mRNA and protein) and TFAM (only mRNA) expression levels when compared to HFD fed rats (Figure 5A,B).

Mitochondrial DNA content was measured in experimental heart tissues by isolating mitochondrial DNA. We found a significant decrease in cardiac mitochondrial DNA content in HFD fed rats. The administration of SRT1720, Oroxylin-A, and combination of SRT1720 and Oroxylin-A significantly (*p* < 0.05) improved cardiac mitochondrial DNA content when compared to HFD fed rats. Whereas, metformin treatment in high fructose diet-fed rats did not improve mitochondrial DNA content significantly (*p* < 0.05) (Figure 5C). The same was confirmed by an in-vitro experiment where we induced insulin resistance in cardiomyoblast cells (H9c2) by treating them with palmitic acid (PA) and measured the mitochondrial content using mito-tracker green dye. We found a significant decrease in mitochondrial content in palmitate treated H9c2 cells. However, sirtuin activators SRT1720 and Oroxylin-A and their combination significantly (*p* < 0.05) improved the mitochondrial content in H9c2 cells (Figure S4).

### 3.10. Sirtuin Activation Improves Cardiac Mitochondrial DNA Encoded ETC Complex Gene and Protein Expression, and Their Activity in High Fructose Diet-Fed Rats

**Figure 6:**
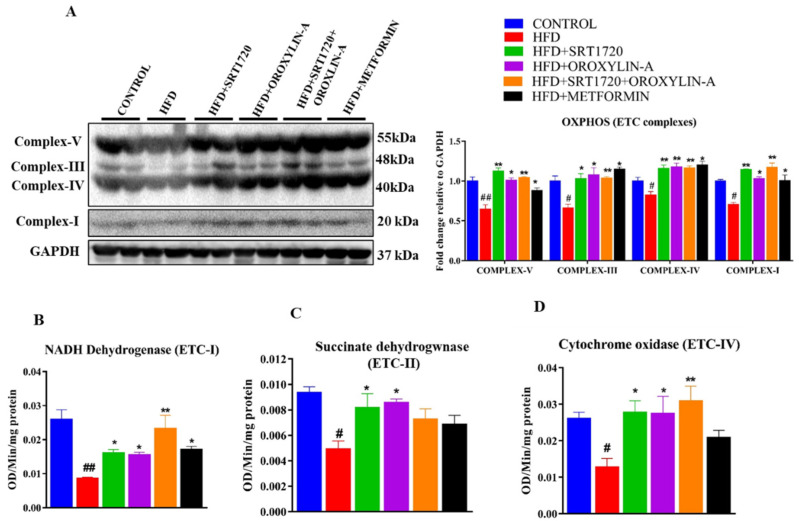
Sirtuin activation enhances ETC complex (OXPHOS) protein expression and activity in pre-diabetic rats (**A**) OXPHOS protein expression. (**B**) ETC complex-1 (NADH dehydrogenase activity). (**C**) ETC complex-II (Succinate dehydrogenase) activity. (**D**) ETC complex-IV (cytochrome-c oxidase) activity. Data was represented as Mean ± SEM, ^#^ *p* < 0.05 vs. Control, ^##^ *p* < 0.01 vs. Control, * *p* < 0.05 vs. HFD, ** *p* < 0.01 vs. HFD, (*n* = 4) for western blotting, (*n* = 5) for mRNA expression and enzymatic activity.

We measured the expression of mitochondrial DNA encoded gene (mRNA) levels by qPCR and protein expression by immunoblotting. We observed that the mRNA and protein expression levels of mitochondrial DNA encoded genes were significantly (*p* < 0.05) decreased in the heart of HFD fed rats. Sirtuin activation with SRT1720, Oroxylin-A, and their combination significantly (*p* < 0.05) increased the ETC complex gene and protein expression. Administration of metformin along with high fructose diet also improved mitochondrial DNA encoded gene and protein expression in HFD fed rats (Figure S6 and Figure 6A).

Then we measured the mitochondrial ETC complexes (i.e., ETC-I: NADH-dehydrogenase, ETC-II: Succinate dehydrogenase and ETC-IV; cytochrome oxidase) activity. We found that a significant (*p* < 0.05) decrease in ETC complexes activity in the heart of HFD fed rats. However, the affected ETC complex activity due to high fructose diet was significantly (*p* < 0.05) improved by administration of SRT1720, Oroxylin-A and combination of SRT1720 and Oroxylin-A. However, the administration of metformin improved only ETC protein expression but did not improve the ETC complex proteins activity (Figure 6B–D). Citrate synthase, an enzyme of the TCA cycle, was significantly (*p* < 0.05) decreased in the heart of HFD fed rats. Administration of Oroxylin-A and combination of SRT1720 and Oroxylin-A improved citrate synthase activity. However, we did not find any significant change in β-hydroxy acyl CoA dehydrogenase (an enzyme essential for fatty acid oxidation) levels in any of the groups studied (Figure S5).

### 3.11. Sirtuin Modulation Improved Mitochondrial Membrane Potential Andoxygen Consumption Rate in Palmitate-Induced Insulin Resistance Cardiomyoblast Cells

**Figure 7:**
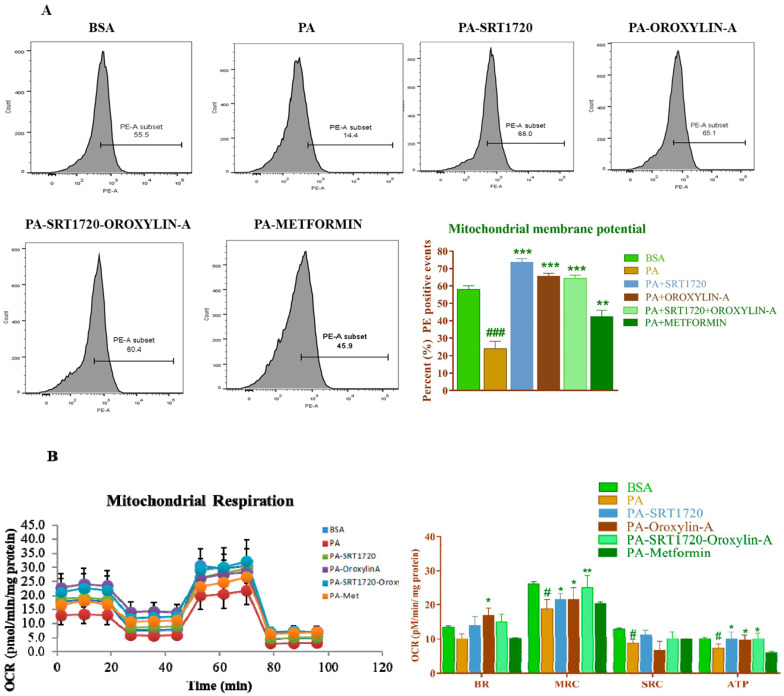
Sirtuin activation improves mitochondrial membrane potential and oxygen consumption rate in palmitate-induced insulin-resistant cardiomyoblast cells: (**A**) Effect of Sirtuin activation on mitochondrial membrane potential. (**B**) Effect of Sirtuin activation on mitochondrial oxygen consumption rate (BR; Basal respiration, MRC; Maximal respiration capacity, SRC; Spare respiratory capacity, ATP; ATP-linked oxygen consumption rate.). Data was represented as Mean ± SEM, ^#^ *p* < 0.05 vs. BSA, ^###^ *p* < 0.001 vs. BSA, * *p* < 0.05 vs. PA, ** *p* < 0.01 vs. PA, *** *p* < 0.001 vs. PA, (*n* = 3).

We evaluated the effect of sirtuin modulation on palmitate (PA)-induced mitochondrial dysfunction in cardiomyoblast cells. We measured the mitochondrial membrane potential and mitochondrial oxygen consumption rate in palmitate-induced insulin resistance cardiomyoblast cells. We observed a significant (*p* < 0.01) decrease in mitochondrial membrane potential and oxygen consumption rate in palmitate treated (PA) cells. However, palmitate-induced mitochondrial dysfunction was significantly precipitated with sirtuin activators, SRT1720, Oroxylin-A and the combination improved mitochondrial membrane potential oxygen consumption rate and most of the mitochondrial functional parameters (maximal respiratory capacity and ATP-linked oxygen consumption rate). None of the mitochondrial functional parameters were significantly improved after metformin treatment (Figure 7A,B).

### 3.12. Sirtuin Activation Improves Cardiac Antioxidant Enzyme Expression and Activity and Reduces Oxidative Damage in High Fructose Diet-Fed Rats

**Figure 8:**
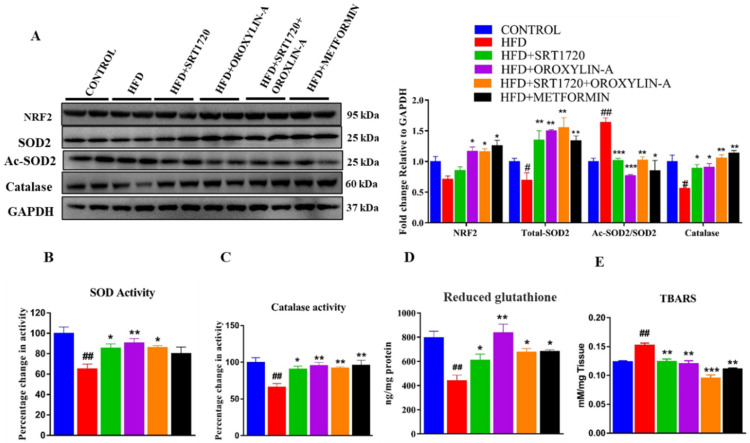
Sirtuin activation reduces high fructose diet-induced oxidative stress in SD rats. (**A**). NRF-2 and Antioxidant (SOD2, and catalase) protein expression. (**B**) SOD activity. (**C**) Catalase activity. (**D**). Reduced glutathione (GSH) levels. (**E**) TBARS levels. Data was represented as Mean ± SEM, ^#^ *p* < 0.05 vs. Control, ^##^ *p* < 0.01 vs. Control, * *p* < 0.05 vs. HFD, ** *p* < 0.01 vs. HFD, *** *p* < 0.001 vs. HFD, (*n* = 4) for western blotting, (*n* = 5) for mRNA expression and enzymatic activity (Blotts were developed different gels due to close molecular weight of the protein of interest, an equal amount of the proteins were loaded into the gels).

Cardiac oxidative stress was evaluated by measuring the expression and activity of different antioxidant enzymes and transcription factor NRF2. We found that a significant (*p* < 0.05) decrease in mitochondrial antioxidant enzyme expression in HFD fed rats when compared to control rats. However, administration of SRT1720, Oroxylin-A and combination of SRT1720 and Oroxylin-A from 12th to 20th week significantly (*p* < 0.05) improved the expression and activity of antioxidant enzymes (SOD2 and Catalse). Oral administration of metformin also improved the antioxidant enzyme expression but no change in SOD and catalase activity when compared to HFD fed rats. We also measured the ratio of Ac-SOD2/SOD2 in HFD fed rat hearts and found a significant increase in the Ac-SOD2/SOD2 ratio than control rats. Sirtuin activation with SRT1720, Oroxylin-A, and combination of SRT1720 and Oroxylin-A significantly reduced Ac-SOD2/SOD2 in high fructose diet fed rat heart (Figure 8A–C). While NRF2 levels were not changed significantly in HFD fed rat hearts, Oroxylin-A and Metformin treatment along with high fructose diet enhanced NRF2 levels when compared to HFD fed rats (Figure 8A).

To assess the oxidative damage in high fructose diet fed animals, we measured cardiac reduced glutathione (GSH) and TBARS levels, a marker of lipid peroxidation. We found a significant (*p* < 0.01) increase in cardiac TBARS levels in HFD fed rat heart tissues when compared to control diet-fed rats. Sirtuin activation with SRT1720, Oroxylin-A, and their combination of SRT1720 and Oroxylin-A, and standard drug metformin treatment significantly (*p* < 0.01) decreased raised cardiac TBARS levels (Figure 8E). We found a significant decrease in the reduced form of glutathione (GSH) in HFD fed rats. Sirtuin activation with SRT1720, SRT1720+Oroxylin-A, and standard drug metformin significantly (*p* < 0.05) improved GSH levels when compared to HFD fed rats, and this was more prominent (*p* < 0.001) in Oroxylin-A treated groups when compared to other groups (Figure 8D).

### 3.13. Effect of Sirtuin Activation on Palmitate-Induced Oxidative Stress in Rat Cardiomyoblast Cells

**Figure 9:**
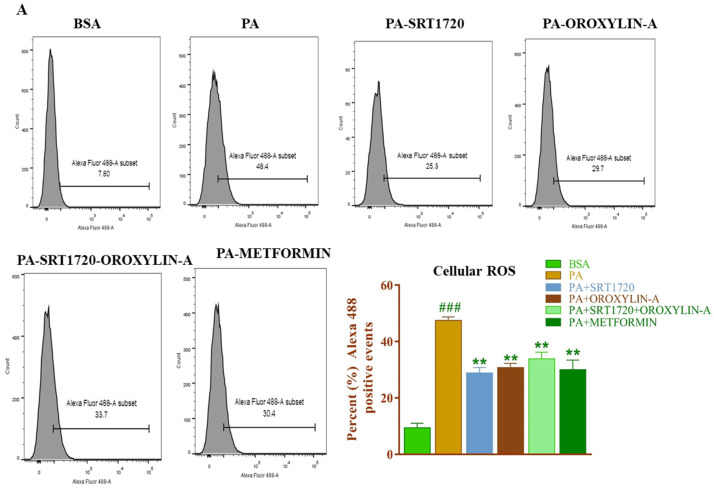

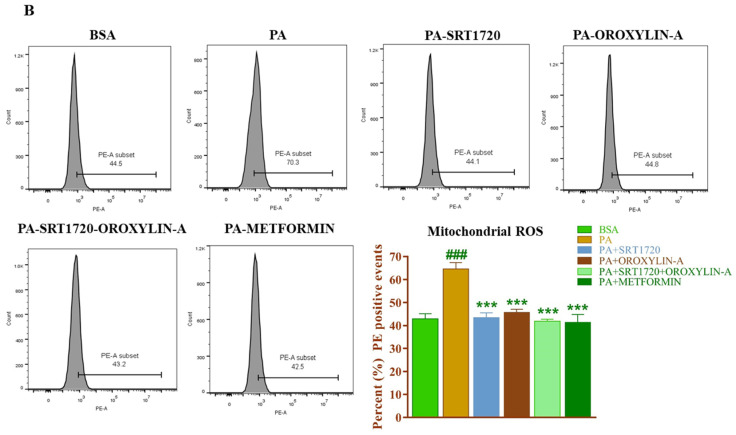
Sirtuin activation reduces the palmitate-induced cellular and mitochondrial ROS production in cardiomyoblast cells. (**A**) Effect of sirtuin modulation on palmitate-induced cellular reactive oxygen species. (**B**) Effect of sirtuin modulation on palmitate-induced mitochondrial reactive oxygen species. Data was represented as Mean ± SEM, ^###^ *p* < 0.001 vs. BSA, *** *p* < 0.001 vs. PA ** *p* < 0.01 VS PA (*n* = 3).

To further confirm the effect of sirtuin modulation on oxidative stress in insulin-resistant cardiomyoblast cells, we induced insulin resistance in H9c2 cells with palmitic acid (PA) and measured the cellular and crude mitochondrial reactive oxygen species in the insulin-resistant cardiomyoblast (H9c2) cells. As we did not measure the NADH, ascorbic acid and GSH levels in insulin-resistant H9c2 cells, measurement of mitochondrial reactive oxygen species was an indirect method to measure cellular oxidative stress. We observed a significant increase in cellular and crude mitochondrial reactive oxygen species in PA treated H9c2 cells. Sirtuin modulators’ treatment significantly (*p* < 0.05) attenuated palmitate-induced both cellular and mitochondrial reactive oxygen species generation. Administration of metformin a standard drug also able to reduce oxidative stress in cardiomyoblast cells effectively (Figure 9A,B).

### 3.14. Sirtuin Activation Promotes Mitochondrial Fusion in High Fructose Diet-Fed Rats

We assessed the mitochondrial fusion and fission related protein expression by western blotting in HFD fed rats. We found a significant (*p* < 0.05) decrease in one of the mitochondrial fusion (Mfn2) protein expression in HFD fed rats when compared to control diet-fed rats. Mfn2 protein expression was significantly (*p* < 0.05) increased upon sirtuin activation and standard drug metformin treatment. However, other mitochondrial fusion proteins i.e., Mfn1 and Opa1 and mitochondrial fission protein, Drp1expression were not affected in any of the groups (Figure S7).

### 3.15. Sirt1 Modulation Regulates the Mitochondrial Biogenesis-Related Gene and Protein Expression in Cardiomyoblast Cells

**Figure 10:**
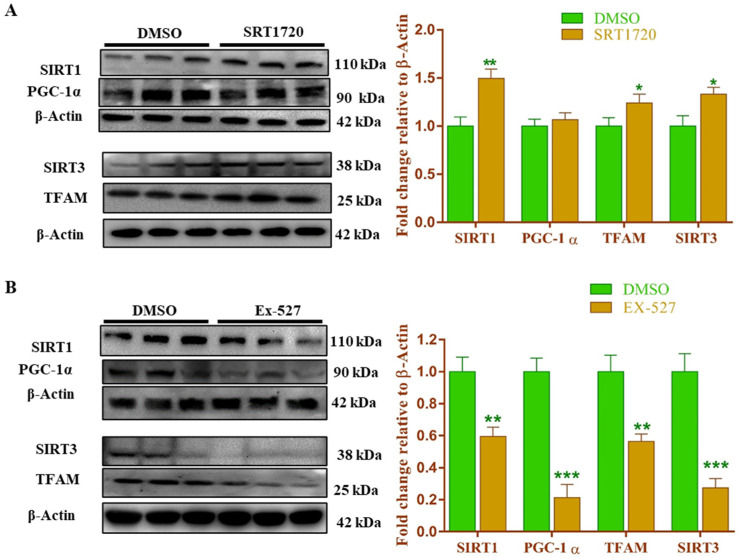
Sirt1 modulation regulates mitochondrial biogenesis through Sirt3 dependent manner in rat cardiomyoblast (H9c2). (**A**) Mitochondrial biogenesis-related protein expression in Sirt1 activation condition. (**B**) Mitochondrial biogenesis-related protein expression in Sirt1 activation condition. Data was represented as Mean ± SEM (*n* = 3), * *p* < 0.05 vs. DMSO, ** *p* < 0.01 vs. DMSO, *** *p* < 0.001 vs. DMSO.

We wanted to know the effect of Sirt1 modulation on mitochondrial biogenesis in rat cardiomyoblasts (H9c2) cells. We treated H9c2 cells with SRT1720 as a Sirt1 activator, and measured sirtuins (Sirt1 and Sirt3) and the mitochondrial biogenesis related (PGC-1α, and TFAM) mRNA and protein expression. We found that Sirt1 activation significantly (*p* < 0.05) increased Sirt1, Sirt3 and TFAM mRNA and protein expression when compared to vehicle (DMSO), treated H9c2 cells (Figure 10A and Figure S8A). We further confirmed the role of Sirt1 on cardiomyoblasts mRNA and protein expression related to mitochondrial biogenesis genes after Ex527 (Sirt1 inhibitor) treatment. We found a significant (*p* < 0.05) decrease in Sirt1, Sirt3, PGC-1α and TFAM gene and protein expression when compared to vehicle (DMSO) treated cells (Figure 10B and Figure S8B).

## 4. Discussion

Diabetes mellitus is a complex metabolic disorder and associated with several complications. Cardiovascular complications are responsible for the major proportion of death in diabetes. Several molecular mechanisms like insulin resistance, altered metabolism, oxidative stress, lipotoxicity, ER-stress, apoptosis, and mitochondrial dysfunction are involved in developing diabetes-associated cardiovascular complications [24]. Mitochondrial dysfunction plays a crucial role to develop cardiac complications in type-2 diabetes. Currently, there is no specific treatment for diabetes-associated cardiac complications by targeting mitochondrial dysfunction. Antidiabetic, lipid-lowering, and antihypertensive drugs were used as an adjuvant therapy for diabetes-associated cardiac complications [25]. Several targets are being explored to reduce cardiac disorder in diabetes. Sirtuins, a group of deacetylases, are potential targets that can regulate cellular metabolism, oxidative stress, and mitochondrial health. Nuclear sirtuins Sirt1 and Sirt6 play an important role in the regulation of metabolic regulation by monitoring the nuclear protein acetylation levels. Data showed that sirtuins, specially Sirt1 and Sirt3 activation can prevent/reverse the progression of several chronic metabolic diseases through the regulation of multiple histone and non-histone proteins function by regulating their acetylation status. Sirt1 regulates cellular metabolism by deacetylating several nuclear transcription factors and histone proteins, and thereby modulates the transcription of several genes which involved in metabolism and mitochondrial homeostasis [7,8,26]. While Sirt3, majorly restricted to mitochondria, deacetylates mitochondrial proteins and regulates oxidative phosphorylation, TCA cycle, fatty acid metabolism and mitochondrial dynamics. In the present study, we aimed to investigate the protective effect of sirtuin modulators in diabetes-associated cardiac complications. We looked at the beneficial effects of SRT1720, a Sirt1 activator [27,28,29,30], Oroxylin-A, a Sirt3 activator [31,32,33], and their combination in insulin resistance/pre-diabetic rat heart.

As Sirt1 and Sirt3 play an essential role in metabolic regulation [30,34,35,36], we were interested to evaluate the effect of Sirt1 and Sirt3 activation and their combination on fasting blood glucose levels and insulin resistance. A significant decrease in fasting blood glucose levels and insulin resistance was observed after treatment with Sirt1 and Sirt3 activators. To further confirm the effect on insulin resistance, we measured the serum insulin and calculated HOMA-IR. We found increased serum insulin levels and HOMA-IR in high fructose diet-fed rats. These parameters were decreased with sirtuin modulators and standard drug treatment. Although Sirt1 and Sirt3 activation able to reduce insulin resistance, we could not observe any additional benefit with the combination. We did mechanistic study to understand the effect of siutuins modulators on cardiac insulin resistance and measured glucose uptake in H9c2 cells. Palmitate induced insulin resistance in H9c2 cells was reduced by the treatment of SRT1720, Oroxylin-A, and their combination. After confirming the Sirt1 and Sirt3 activation on systemic and cardiac insulin resistance, we wanted to know the effect of Sirt1 and Sirt3 activation on lipid parameters i.e., triglycerides, LDL, HDL, serum free fatty acids and total cholesterol in pre-diabetic rats fed with high fructose diet. We observed that sirtuin activation reduces the high fructose diet-induced dyslipidemia in rats. While Sirt1 and the combination were effective to reduce most of the lipid parameters, Sirt3 activation normalized only serum triglycerides, free fatty acids and increases HDL levels.

As insulin resistance affects cardiac physiology and function [37,38,39,40,41], we were keen to know the effect of high fructose diet-induced insulin resistance on electrocardiography and echocardiography parameters, and the role of sirtuin activation in pre-diabetic rats. We measured the electrocardiography at the 12th week before starting the therapy and 20th week, at the end of study. We did not observe any significant change in the heart rate, RR-interval, QT-interval, and QTc-interval at 12th-week time point. However, we observed a significant change in the heart rate and RR-interval at 20th-week. The administration of sirtuin (Sirt1, Sirt3 and their combination) activators significantly improved electrocardiography of the diabetic rats and our results were supported by previously literature [26,42]. The change in electrophysiological parameters that we observed in diabetic heart might be due to some defects in the activity and expression level of sarcolemmal enzymes and ion channels. Na^+^/K^+^ ATPase activity, ATP production, and mitochondrial and cytosolic Ca^2+^ levels in cardiomyocyte. These changes plays vital role in the generation of action potential and myocardium contraction in diabetic and ischemic heart disease conditions [43,44,45]. To understand the myocardial structure and function in insulin resistance rats, we assessed the cardiac function and structure by 2D echocardiography at the 12th and 20th week. We did not observe any significant changes in the functional parameters (ejection fraction and fractional shortening) of heart by 12 weeks of high fructose diet feeding. However, we observed a significant change in structural parameters of diabetic rat heart i.e., increased thickness of the left ventricular posterior wall. Cardiac hypertrophy in high fructose diet-fed rats was also correlated with increased heart weight to body weight ratio and increased QTc intervals at 12th week. Cardiac hypertrophy of the diabetic rat heart was further supported by measuring the fetal cardiac hypertrophic gene expression. We found a significant increase in β-MHC mRNA expression levels in high fructose diet-fed rats, and administration of SRT1720, Oroxylin-A, and combination showed a reduction in β-MHC mRNA expression. Interestingly, we did not observe any hypertrophy in the heart from rats fed with high-fructose diet for 20 weeks. The reason behind not observing the cardiac hypertrophy at 20 weeks could be due to the cardiomyocyte death due to induction of apoptosis. Similar findings were also supported by previous studies [44,45,46,47,48,49]. Although, we did not observe any significant change in the body weight of high fructose diet fed rats, it was decreased significant in SRT1720 treated rats. This result can be explained by the fact that increased Sirt1 activity due to SRT1720 may cause calorie restriction in prediabetic rats. High fructose diet feeding of 20 weeks significantly increased left ventricular lumen diameter during systole (LVDs) and a significant decrease in fractional shortening and ejection fraction. However, sirtuin activators SRT1720, oroxylin-A, and the combination improved cardiac structural and functional parameters in high fructose diet-fed rats similar to standard drug metformin. Although, we observed a significant improvement of cardiac structure and function by Sirt1 and Sirt3 modulators, no additional benefit was noticed with the combination.

Previous literature reported that high fructose diet can increase blood pressure in rats [46,47]. Similarly, we found a significant increase in systolic blood pressure after 12 weeks of high fructose diet feeding. 20-weeks of high fructose feeding further increased the systolic and mean arterial blood pressure. These parameters were significantly reduced with the administration of SRT1720, Oroxylin-A, and their combination. High blood pressure and insulin resistance together can promote the remodeling of cardiac tissue and enhances cardiac fibrosis [16,48]. Similar to earlier studies, chronic feeding of high fructose diet in rats also caused cardiac fibrosis as represented by perivascular and interstitial collagen deposition. Sirtuin activators SRT1720, oroxylin-A, and the combination prohibited the cardiac fibrosis effectively. Metformin, a standard drug also decreased cardiac fibrosis in high fructose diet-fed pre-diabetic rats. The fibrosis in the pre-diabetic heart was also correlated with the profibrotic gene (MMP9, and Collagen-I) expression, which was further reduced after treatment with sirtuin modulators.

As reported earlier [16,40], the present study observed a significant decrease in the expression and activity of Sirt1 and Sirt3 in 20-weeks high fructose diet-fed rats. However, the Sirt1 activity was improved only in SRT1720 treated group but not in combination group. This could be due to off-target effect of Oroxylin-A or drug interaction. Sirt3 expression and activity were significantly improved in both Sirt1 and Sirt3 activation groups. The possible reason for this observation was due to presence of Sirt3 as a downstream of Sirt1. We expected that enhanced Sirt1 and Sirt3 activity in the heart could be beneficial to improve cardiac function via improving mitochondrial health.

Mitochondrial dysfunction is the key factor in type-2 diabetes for developing diabetes-associated cardiac complications [16,49,50,51,52,53,54]. In our study, we found that mitochondrial biogenesis was significantly affected by chronic high fructose diet feeding by suppressing the PGC-1α, a master regulator of mitochondrial biogenesis. However, Sirt1, Sirt3, and their combination significantly improved mitochondrial biogenesis by up-regulation of mitochondrial biogenesis related gene expression. Expression of PGC-1α in Oroxylin-A treated animals was significant when compared to SRT1720 treated group. This could be due to the positive feedback mechanism of Sirt3 and PGC-1α. We observed similar results in an in-vitro experiment, a significant increase in PGC-1α expression in H9c2 cells after Sirt3 overexpression or Oroxylin-A treatment. Similarly, a significant decrease in PGC-1α expression was observed in Sirt3 knockdown H9c2 cells. This observation and some other reports [55,56] strongly suggest us that there is a possibility of an existing positive feedback mechanism between Sirt3 and PGC-1α expression. Increased mitochondrial biogenesis was further confirmed by measuring the mitochondrial DNA content in both in-vivo animal and in-vitro H9c2 cells. Interestingly, we found a significant decrease in mitochondrial DNA content and mitochondrial content in the hearts of high fructose diet fed animals. and palmitic acid-treated H9c2 cells respectively. Activation of Sirt1 and Sirt3 able to increase the mitochondrial DNA content both in-vivo and mitochondrial content in an in-vitro experiment with H9c2 cells. This indicates that Sirt1 and Sirt3 activation play an important role to enhance mitochondrial biogenesis in the diabetic rat heart. Mitochondrial DNA encodes 13 mitochondrial electron transport chain (ETC) complex subunits, which assemble with nuclear-encoded proteins to form mitochondrial ETC complexes [57,58,59,60,61,62,63]. We measured the mitochondrial DNA encoded gene and OXPHOS protein expression. High fructose diet significantly downregulates the mitochondrial DNA encoded gene and protein expression, and their activity in rat hearts. However, Sirt1, Sirt3, and the combination significantly improved mitochondrial function by enhancing the mitochondrial DNA encoded gene expression and OXPHOS protein expression. We further measured the important mitochondrial enzymes activity like citrate synthase and β-hydroxy acyl CoA dehydrogenase activity to assesses the mitochondrial function of diabetic rat heart. A significant decrease in citrate synthase activity was observed in the diabetic rat hearts. However, activation of sirtuins significantly improved the citrate synthase activity. We did not observe any significant changes in β-hydroxy acyl CoA dehydrogenase activity in diabetic rat heart as well as other treatment groups.

To further confirm the role of Sirt1 and Sirt3 activators (SRT1720 and Oroxylin-A) on mitochondrial health and function, we measured the mitochondrial membrane potential and function in an in-vitro experiment on palmitate-induced insulin-resistant H9c2 cells. We observed a significant decrease in mitochondrial intermembrane potential and oxygen consumption rate and functional parameters of mitochondria (basal respiration, maximal respiratory capacity, spare respiratory capacity, and ATP-linked oxygen consumption rate) in palmitic acid-treated cells. SRT1720 and Oroxylin-A treatment enhanced mitochondrial membrane potential, oxygen consumption rateand function in palmitate-treated H9c2 cells.

Mitochondrial function depends on their dynamics i.e., mitochondrial fusion and fission, and thus play a crucial role to provide energy to the cardiac muscle [54,55,56,57,58,59,60,61]. During stress conditions, small mitochondria fuse to form an efficient giant mitochondrial network for generating higher levels of ATP. On the other hand, a large mitochondria can fragment into small non-functional mitochondria in chronic stress conditions, known as mitochondrial fission. In the present study, we measured the expression of mitochondrial fusion (Mfn1, Mfn2, and Opa1) and fission (Drp1) regulatory proteins. We observed decreased mitochondrial fusion as observed by reduced expression of Mfn2 protein levels in high fructose diet-fed rat hearts. Activation of Sirt1, Sirt3, and their combination enhanced the high fructose diet-induced suppression of Mfn2 expression. However, metformin did not increase the Mfn2 expression in high fructose diet-fed animals.

Decreased mitochondrial fusion can enhance more inefficient mitochondria in cells and responsible for reactive oxygen species (ROS) generation. This increased ROS may cause oxidative stress-induced cardiac complications and cardiac remodeling [56,57]. We have previously reported the cardioprotective effect of garlic and resveratrol against diabetes-induced oxidative stress via activation of Sirt1 and Sirt3, respectively [16,51]. In the present study, we measured the expression of nuclear respiratory factor-2 (NRF-2), the upstream regulator of endogenous antioxidants. We have also measured endogenous antioxidants like SOD2 and catalase, and glutathione (GSH), and TBARS, a marker of oxidative stress in high fructose diet-fed rat hearts. Although we did not find any change in NRF2 levels in any group, but we observed reduced cardiac expression and activity of antioxidant enzymes like SOD2 and catalase in high fructose diet-fed rats. SRT1720 and Oroxylin-A administration improved the expression and activity of SOD2 and catalase. The low levels of reduced glutathione and increased levels of TBARS in the heart of high fructose diet-fed rats represent increased oxidative damage of diabetic rat heart. We observed more prominent increase in reduced glutathione levels in Oroxylin-A treated animals. This could be due to increased Sirt3 activation and enhanced mitochondrial antioxidant enzyme activity in mitochondria when compared to the other groups. Oroxylin-A treated heart due to presence of higher levels of antioxidant enzymes generates less reactive oxygen species and therefore, reduces the consumption of glutathione. After sirtuin activation, these parameters were improved significantly. To further support our in-vivo study findings, we measured the cellular and mitochondrial specific ROS in palmitate-induced insulin resistance cardiomyoblast cells. Our in-vitro study also correlated with in-vivo findings. A significant increase in cellular and mitochondrial specific ROS that was found in palmitate-induced insulin resistance cardiomyoblast cells was suppressed with SRT1720 and Oroxylin-Atreatment. This data confirmed the role of Sirt1 and Sirt3 activation on mitigating oxidative stress in diabetic rat heart.

Most of the parameters we studied did not show any additional benefit by the administration of SRT1720 and Oroxylin-A together in high fructose diet-induced pre-diabetic rats. However, it should be noted that Oroxylin-A, a Sirt3 activator, is not very specific and has several off-target effects. Our literature search as well as our present animal data indicated that Sirt1 activation independently can regulate Sirt3 expression, activity, and stability in mice models [11,58]. To further confirm, we did an in-vitro experiment where we have activated Sirt1 in rat cardiomyoblast cells and observed the expression of Sirt3 and mitochondrial biogenesis related gene and protein expression. A significant increase in Sirt3 expression, as well as mitochondrial biogenesis related genes and protein (PGC-1α and TFAM) expression, was observed in cardiomyoblast cells after Sirt1 activation. Similarly, Sirt1 inhibition caused a significant decrease in Sirt3 as well as mitochondrial biogenesis related gene and protein expression in cardiomyoblast cells.

## 5. Conclusions

**Figure 11:**
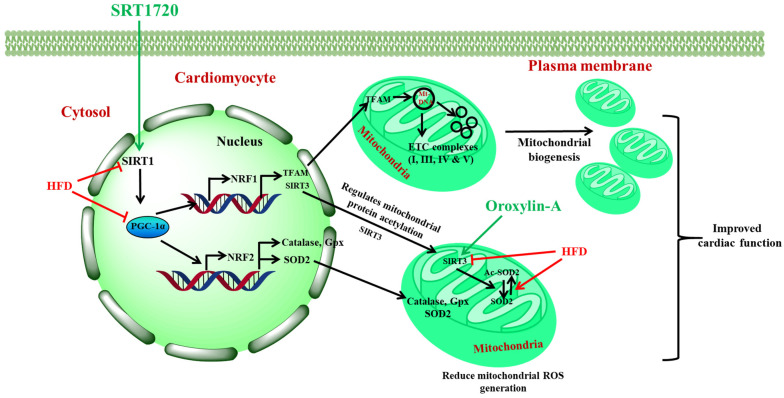
Activation of Sirt1 with SRT1720 can handle the high fructose diet-induced insulin resistance, oxidative stress, and mitochondrial dysfunction through Sirt1-PGC-1α-NRF1- Sirt3-TFAM signaling pathway.

Taken together our results conclude that activation of Sirt1 and Sirt3 can prevent the progression of the disease from diabetes to diabetes-associated cardiac complications by decreasing insulin resistance, oxidative stress, serum lipid profile, and improving mitochondrial function through enhancing mitochondrial biogenesis, OXPHOS expression, and activity. Sirt1 activation alone can improve cardiac complications in the diabetic heart by modulating Sirt3 and improving mitochondrial health as shown in Figure 11. There is no extra benefit for combining Sirt1 and Sirt3 activators for the treatment of diabetes and associated cardiac complication.

## 6. Limitations of the Study

SRT1720 and Oroxylin-A were studied concerning the Sirt1 and Sirt3 activation and their role to maintain cardiac mitochondrial health and cardiac function in diabetic rats. However, both the above molecules are not very specific activators and may have off-target effect. Similarly, we have not measured the levels and activity of Na^+^/K^+^ ATPase, an important sarcolemmal protein, whose level has been reported to be altered in diabetic heart. Inflammation induced by chronic high fructose diet in rats and palmitate in H9c2 cells, and the effect of Sirt1/Sirt3 activator on pro-inflammatory cytokines production in cardiomyocytes was not evaluated. Further study is required to understand more details of the molecular mechanism and application of known sirtuin activator in human diabetic heart.

## Figures and Tables

**Table 1:** Effect of Sirtuin activation on serum lipid profile and free fatty acid levels in diabetic rat.

Parameter	Control	HFD	HFD + SRT1720	HFD + Oroxylin-A	HFD + SRT1720 + Oroxylin-A	HFD + Metformin
Triglycerides. (mg/dL)	81.5 ± 17.85	158 ± 24.2 ^##^	70.5 ± 10.3 ***	52.3 ± 10.3 ***	75.5 ± 31.6 ***	54.0 ± 12.6 ***
Cholesterol (mg/dL)	77.5 ± 4.46	96.3 ± 4.0 ^##^	84.6 ± 7.03 *	9.47 ± 9.7	86.8 ± 12.6 *	97 ± 14.7
LDL (mg/dL)	16 ± 2.92	23.4 ± 2.7 ^#^	18.44 ± 2.0 *	19.8 ± 2.4	18.6 ± 2.3 *	19.2 ± 1.79
HDL (mg/dL)	78.8. ±4.39	78.1 ± 1.6	86 ± 3.2	91.8 ± 3.8 *	79.8 ± 3.8	93.8 ± 5.06 *
Free fatty acid (mM/L)	0.84 ± 0.12	1.38 ± 0.15 ^##^	0.97 ± 0.12 *	0.88 ± 0.07 *	1.08 ± 0.19 *	0.81 ± 0.19 **

Data were represented as Mean ± SEM, ^#^ *p* < 0.05 vs. Control, ^##^ *p* < 0.01 vs. Control, * *p* < 0.05 vs. HFD, ** *p* < 0.01 vs. HFD, *** *p* < 0.001 vs. HFD, (*n* = 6).

## Data Availability

The data used to support the findings of this study are available from the corresponding author upon request.

## Supplementary Materials

The following are available online at https://www.mdpi.com/2076-3921/10/3/338/s1, Figure S1: Twelve weeks of high fructose diet feeding induces cardiac hypertrophy without affecting cardiac functional parameters, Figure S2: Sirtuin activation reduces high fructose diet-induced blood pressure in rats. Figure S3: Sirtuin activation ameliorates high fructose diet-induced cardiac hypertrophy in rats. Figure S4: Sirtuin activation enhances mitochondrial content in palmitate-induced insulin resistant cardiomyoblast (H9c2) cells. Figure S5: Sirtuin activation ameliorates high fructose diet-induced perturbation of mitochondrial enzymatic activity in diabetic rat heart. Figure S6: Sirtuin activation enhances mitochondrial DNA-encoded ETC complex gene expression in high fat diet-induced diabetic rats. Figure S7: Sirtuin activation ameliorates high fructose diet-induced perturbation of mitochondrial dynamics in diabetic rat heart. Figure S8: Sirt1 modulation regulates mitochondrial biogenesis-related genes and Sirt3 in rat cardiomyoblast (H9c2). Table S1: List of primers used for quantitative real-time PCR in our study.Table S2: List of antibodies used for western blotting in our study. Table S3: Shown the composition of diets used in the study.
