# Supplementary material for: Sirt1 and Sirt3 Activation Improved Cardiac Function of Diabetic Rats via Modulation of Mitochondrial Function
Source: Antioxidants (Basel). 2021 Feb 24;10(3):338. doi: 10.3390/antiox10030338 (PMC7996143; doi:10.3390/antiox10030338)
Supplement: Supplementary file 1 [file antioxidants-10-00338-s001.pdf]

SUPPLEMENTARY TABLES

Table S1 List of primers used for quantitative real-time PCR in our study.

S.No.	Gene	Forward primer (5' to 3')	Reverse primer (5' to 3')	T_a (°C)
1	**RPL32**	AGATTCAAGGGCCAGATCCT	CGATGGCTTTTCGGTTCTTA	57
2	**SIRT1**	TGACTTCAGATCAAGAGATGGTAT	TGGCTTGAGGATCTGGGAGAT	58
3	**SIRT3**	GCTGCCAGCAAGGTTCTTAC	CCTTTCCACACCCTGGACTA	57
4	**NRF1**	GCTCTTTGAGACCCTGCTTTC	GTGGAGTTGAGTATGTCCGAGT	56
5	**NRF2**	AACCAGAAGCACACTGAAGG	CCATTTCCGAGTCACTGAAC	57
6	**PGC-1α**	ATGTGTCGCCTTCTTGCTCT	ATCTACTGCCTGGGGACCTT	57
7	**TFAM**	AGCTGATGGGCTTAGAGAAGG	ATTTCCCCTGAGCTGACTCAT	62
8	**Mt-ND1**	AAAGAACCCATACGCCCTCT	GGCTCATCCCGATCATAGAA	57
9	**Mt-ND2**	AAGCCCACGATCAACTGAAG	GTCAGTAGTGGAATGGGGCT	57
10	**Mt-ND6**	TACAACCAACATCCCACCCA	GTTGTCTAGGGTTGGCGTTG	57
11	**Mt-CO-II**	TTCCTCATCAGCTCCCTAGT	GTAGGGAGGGAAGGGCAATT	57
12	**Mt-CO-III**	ATGACCACTAACAGGAGCCC	GTGGTGGCCTTGGTATGTTC	57
13	**Mt-ATP6**	AGCAAACATTACAGCAGGCC	ACAGGCTGACTAGAAGGGTG	57
14	**Mt-ATP8**	GCCACAACTAGACACATCCAC	GGGGTAATGAAAGAGGCAAATAG	57
15	**Mfn1**	GAGGGAAGACCAAATCGACA	CAAAACAGACAGGCGACAAA	58
16	**Mfn2**	AGGAAATTGCTGCCATGAAC	GTCTCTTCTCGGTGCAGGTC	58
17	**Drp1**	TCCCAATTCCATTATCCTTG	TCAATACATCCATGGCATCA	58
18	**Opa1**	AAAATCAGAAAAGCCCTTCC	TTTCGGATCCATGATCTGTT	57
20	**MMP2**	GGGTGGTGGTCACAGCTATT	CGGTGTGCAGTGAAGATTGT	57
21	**MMP9**	CCACCGAGCTATCCACTCAT	GTCCGGTTTCAGCATGTTTT	57
22	**COLLAGEN-I**	CTCAAGAGCGGAGAATAC	ATCTGTCCACCAGTGCTT	54
23	**ANP**	AGCGAGCAGACCGATGAAG	AGCCCTCAGTTTGCTTTTCA	57
24	**BNP**	GACCAAGGCCCTACAAAAGA	CCCAAAGCAGCTTGAACTATG	53
25	**β-MHC**	TGGAGCTGATGCACCTGTAG	ACTTCGTCTCATTGGGGATG	55
26	**β2-microglobulin**	ACTGCTACGTGTCTCAGTTCC	CTCCTTCAGAGTGACGTGTTT	57

Table S2 List of antibodies used for western blotting in our study

**S.No.**	**Name of the antibody**	**Cat. No.**	**Dilution**
1	Sirt1	9475S	1:1000
2	Sirt3	Ab189860	1:1000
3	PGC-1α	Ab191838	1:1000
4	TFAM	Ab131607	1:2500
5	NRF1	69432S	1:1000
6	NRF2	Ab137550	1:1000
7	SOD2	13141S	1:2500
8	Ac-SOD2	Ab137037	1:1000
9	Catalase	Ab52477	1:1000
10	OXPHOS	Ab110413	1:250

11	Mfn1	Ab104274	1:1000
12	Mfn2	Ab50838	1:1000
13	Drp1	8570S	1:1000
14	Opa1	80471S	1:1000
15	GAPDH	8884S	1:5000
16	β-Actin	Ab8227	1:5000

Table S3 Shown the composition of diets used in the study.

**S.No.**	**Name of the ingredient**	**Quantity in Corn starch diet**		**Quantity in High fructose diet**	
		Gm	Kcal	Gm	Kcal
**1**	Casein	200	800	200	800
2	DL-Mthionine	3	12	3	12
3	Corn Starch	650	2600	0	0
5	Fructose	0	0	650	2600
6	Cellulose, BW200	50	0	50	0
7	Corn oil	50	450	50	450
8	Mineral Mix S1001	35	0	35	0
9	Vitamin Mix V1001	10	40	10	40
10	Choline bitartarate	2	0	2	0
11	FD&C Blue dye#1	0.2	0	0	0
12	FD& Red dye#40	0	0	0.2	0

SUPPLEMENTARY FIGURES

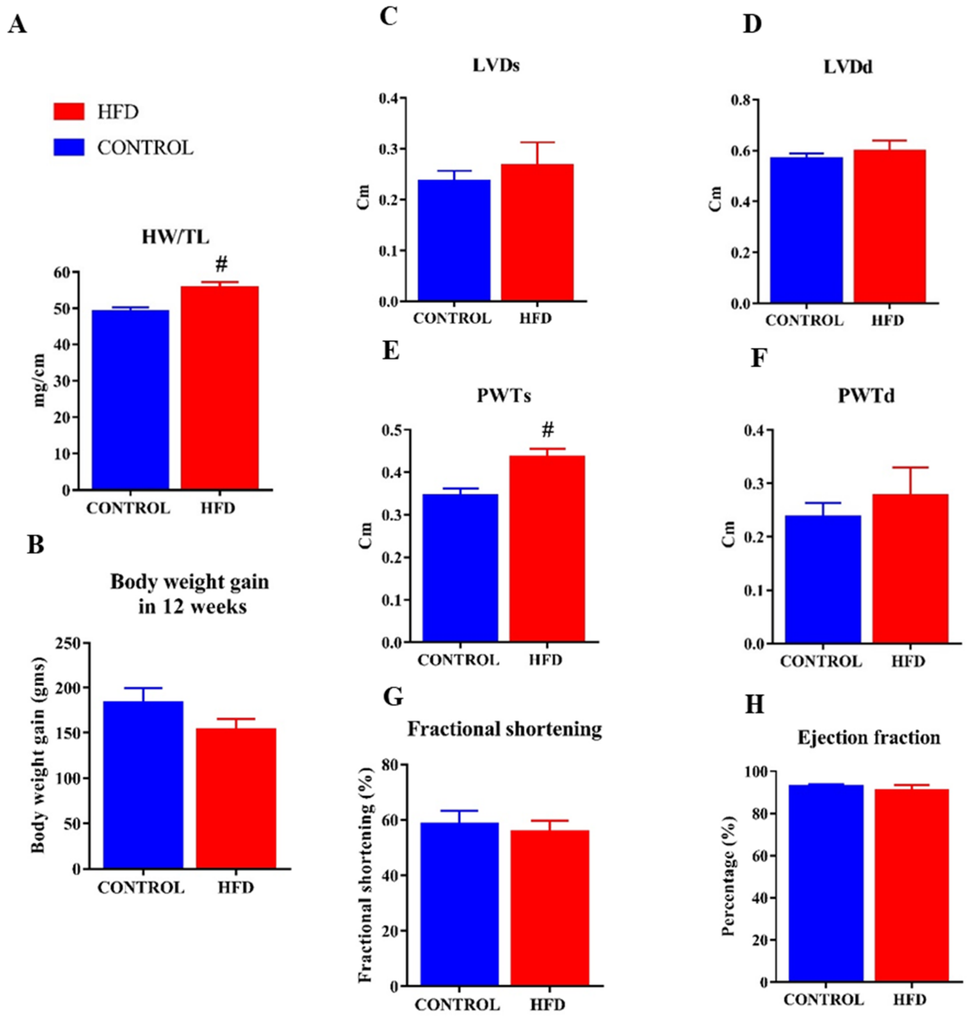

Figure S1. Twelve weeks of high fructose diet feeding induces cardiac hypertrophy without affecting cardiac functional parameters. A Heart weight to body weight ratio. **B** Body weight change. **C** Left ventricular internal diameter during systole (LVD_s). **D** Left ventricular internal diameter during diastole (LVD_d). **E** Posterior wall thickness during systole (PWT_s). **F** Posterior wall thickness during diastole (PWT_d). **G** Fractional shortening. **H** Ejection fraction. Data was represented as Mean ± SEM, #p<0.05 vs Control, ##p<0.01 vs Control, (N=5).

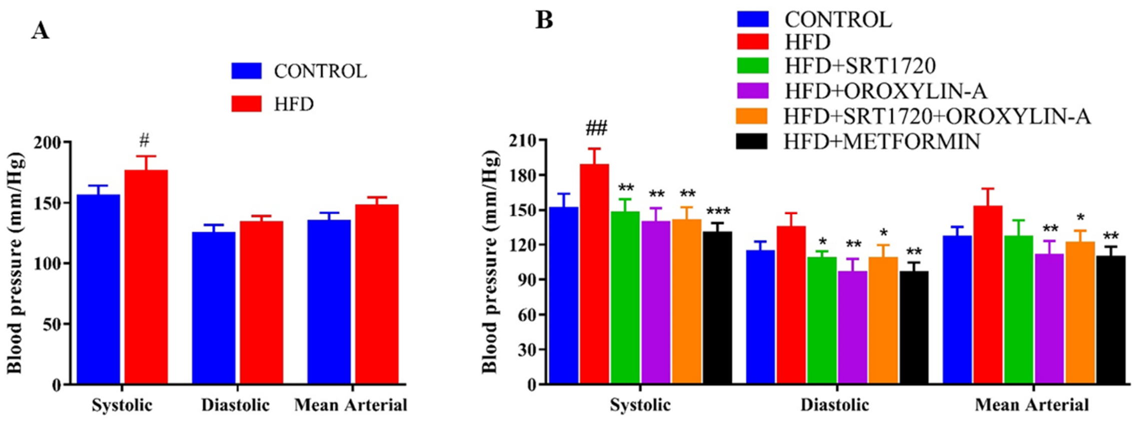

Figure S2 Sirtuin activation reduces high fructose diet-induced blood pressure in rats. A Systolic, diastolic, and mean arterial blood pressure at 12 weeks of high fructose feeding. **B** Systolic, diastolic and mean arterial blood pressure after 8 weeks of treatment with sirtuin activators. Data was represented as Mean ± SEM, #p<0.05 vs Control, ##p<0.01 vs Control, *p<0.05 vs HFD, **p<0.01 vs HFD, ***p<0.001 vs HFD, (N=5).

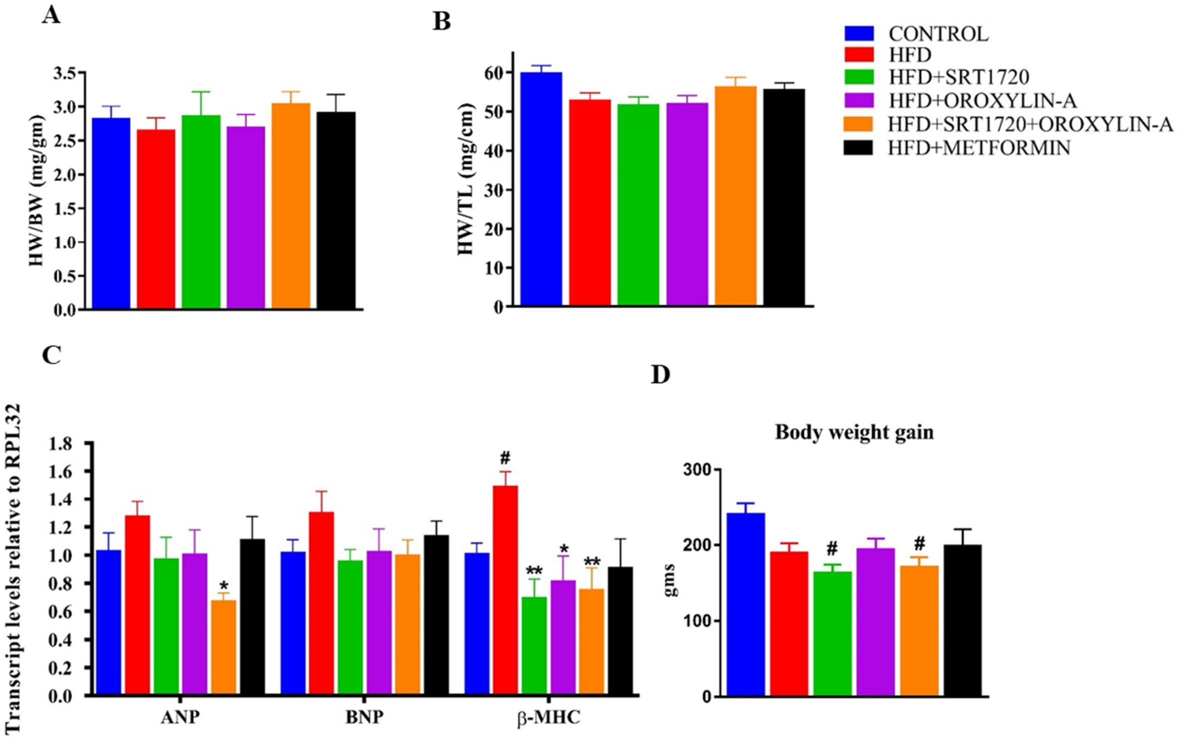

Figure S3 Sirtuin activation ameliorates high fructose diet-induced cardiac hypertrophy in rats. A Heart weight to body weight ratio. **B** Heart weight to tail length ratio. **C** Fetal hypertrophic gene transcripts levels. **D** Body weight change. Data were represented as Mean ± SEM, #p<0.05 vs Control, *p<0.05 vs HFD, **p<0.01 vs HFD, (N=5).

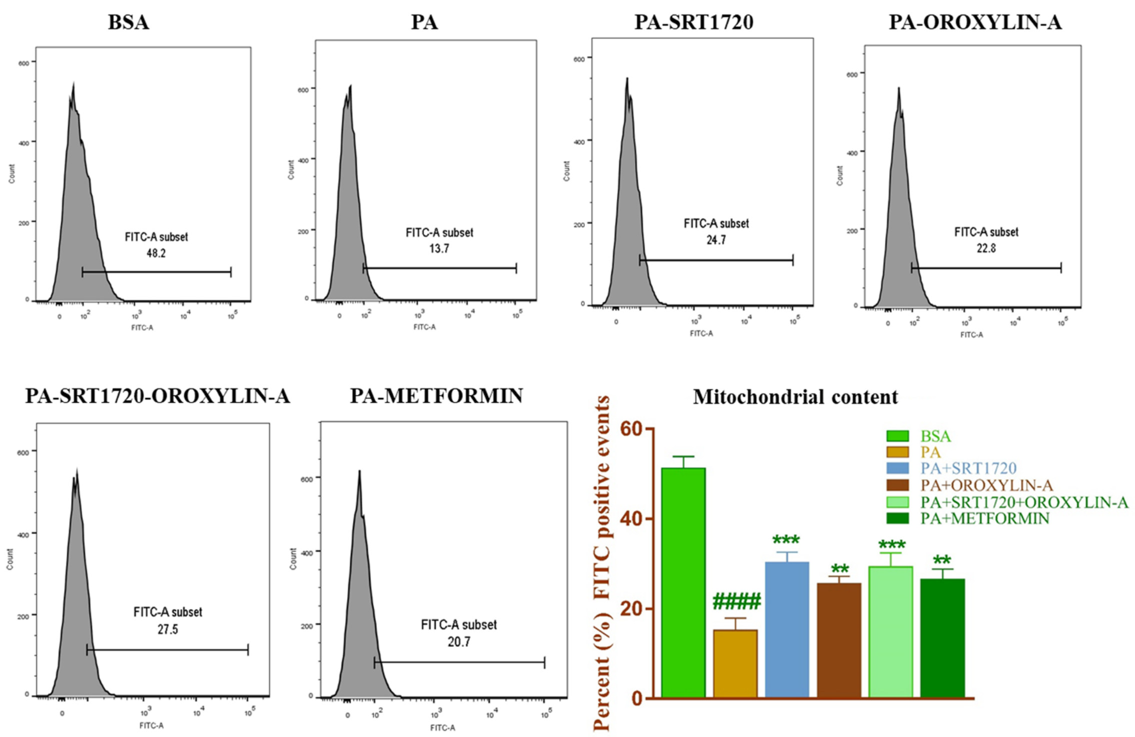

Figure S4 Sirtuin activation enhances mitochondrial content in palmitate-induced insulin resistant cardiomyoblast (H9c2) cells. Effect of sirtuin modulation on mitochondrial content. ###p<0.001 vs BSA, **p<0.01 vs PA, ***p<0.001 vs PA, (n=3).

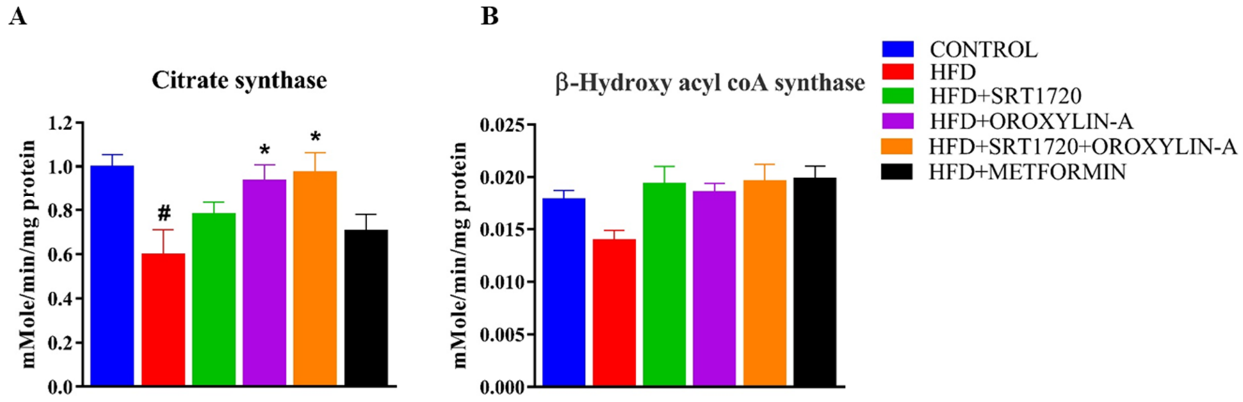

Figure S5 Sirtuin activation ameliorates high fructose diet-induced perturbation of mitochondrial enzymatic activity in diabetic rat heart. A Citrate synthase activity. **B** β-hydroxy acyl CoA dehydrogenase activity. Data was represented as Mean ± SEM, #p<0.05 vs Control, *p<0.05 vs HFD, (N=5).

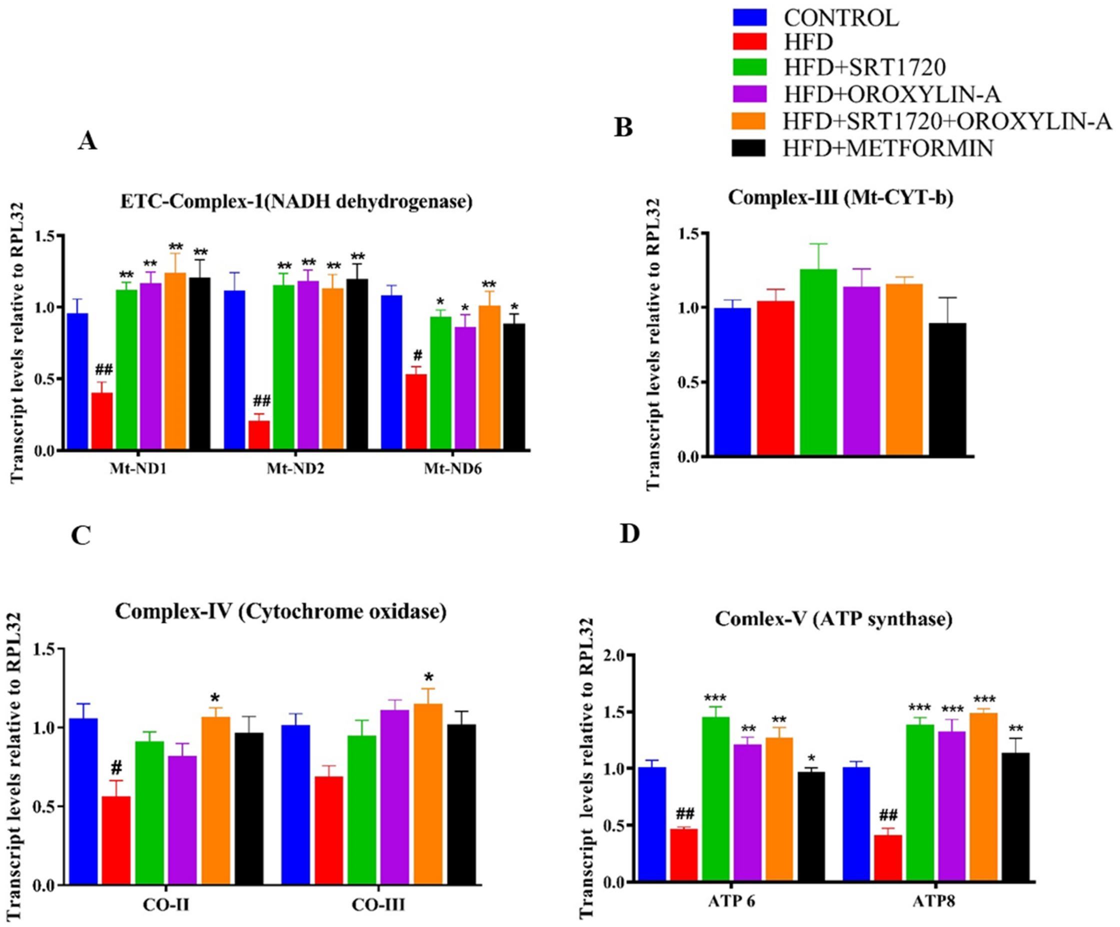

Figure S6 Sirtuin activation enhances mitochondrial DNA-encoded ETC complex gene expression in high fat diet-induced diabetic rats. A ETC complex-I (NADH dehydrogenase) subunit mRNA expression (ND1, ND2, ND6). **B** ETC complex-III (cytochrome reductase) subunit mRNA expression (Cyto-b). **C** ETC complex-IV (Cytochrome oxidase) subunit mRNA levels (Cytochrome oxidase-II and III). **D** ETC complex-V (ATP-synthase) subunit (ATP6 and ATP8) mRNA levels. Data was represented as Mean ± SEM, #p<0.05 vs Control, ##p<0.01 vs Control, *p<0.05 vs HFD, **p<0.01 vs HFD, ***p<0.001 vs HFD, (N=5).

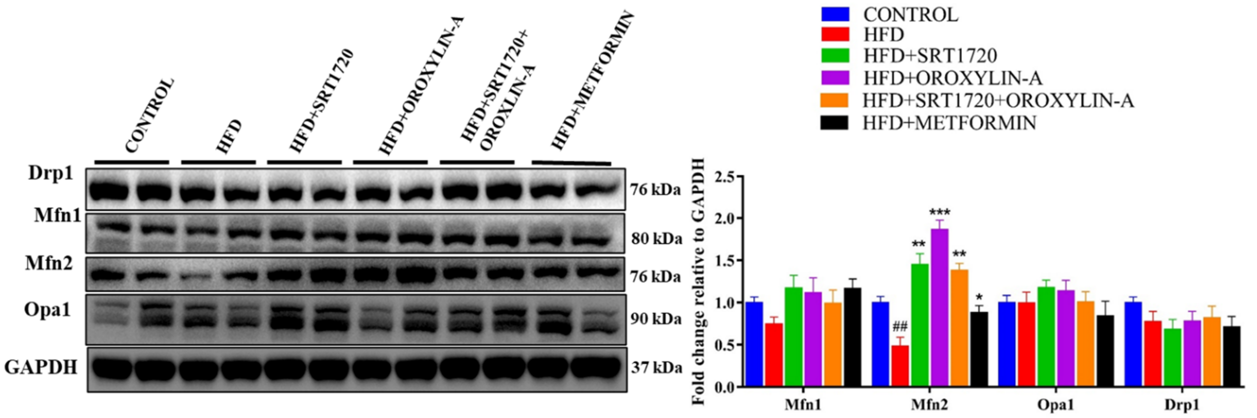

Figure S7 Sirtuin activation ameliorates high fructose diet-induced perturbation of mitochondrial dynamics in diabetic rat heart. A Mitochondrial dynamics-related protein expression. Data was represented as Mean ± SEM, ##p<0.01 vs Control, *p<0.05 vs HFD, **p<0.01 vs HFD, ***p<0.001 vs HFD, (N=4), (Blots were developed from different gels due to same molecular weight equal amount of proteins were loaded into gels).

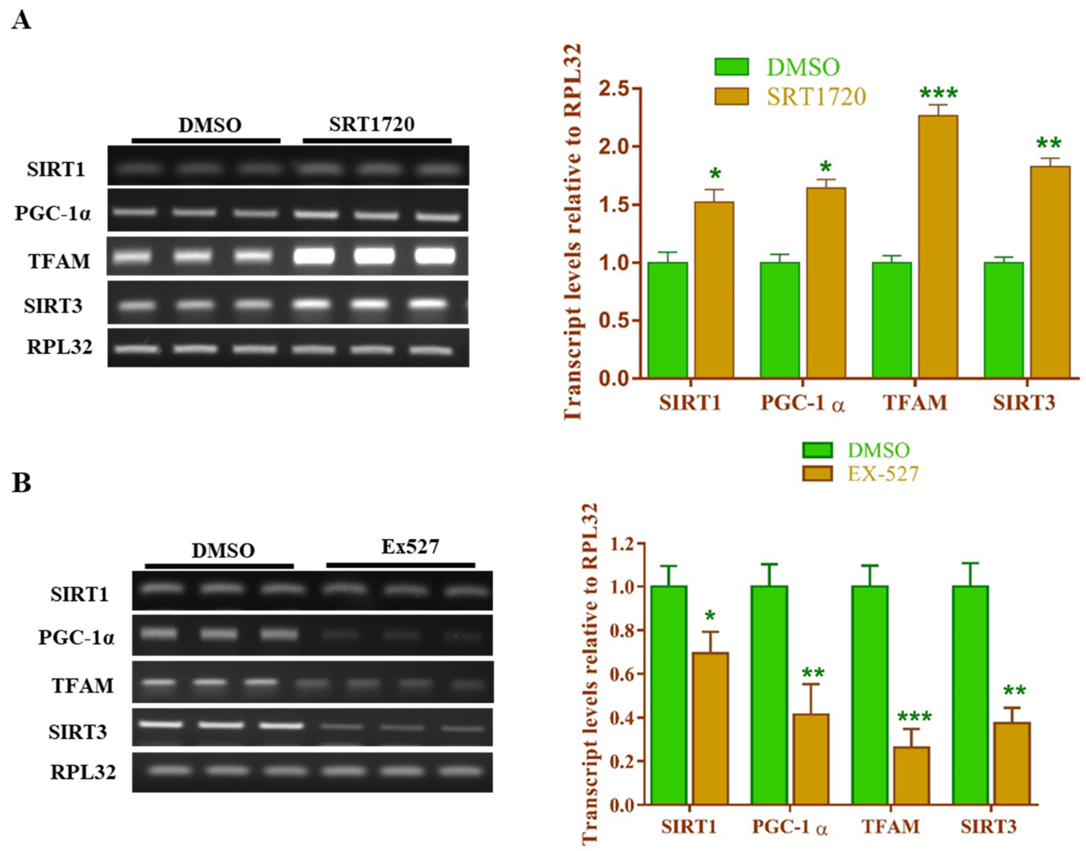

Figure S8 Sirt1 modulation regulates mitochondrial biogenesis-related genes and Sirt3 in rat cardiomyoblast (H9c2). (A) Mitochondrial biogenesis-related gene expression in Sirt1 activation condition. (B) Mitochondrial biogenesis related gene expression in Sirt1 inhibition condition. Data were represented as Mean ± SEM, *p<0.05 vs DMSO, **p<0.01 vs DMSO, n=3.
